# Harnessing the power of microbes: Enhancing soybean growth in an acidic soil through AMF inoculation rather than P-fertilization

**DOI:** 10.1093/hr/uhae067

**Published:** 2024-03-02

**Authors:** Zhongling Wen, Minkai Yang, Aliya Fazal, Hongwei Han, Hongyan Lin, Tongming Yin, Yuelin Zhu, Shouping Yang, Kechang Niu, Shucun Sun, Jinliang Qi, Guihua Lu, Yonghua Yang

**Affiliations:** Institute for Plant Molecular Biology, State Key Laboratory of Pharmaceutical Biotechnology, School of Life Sciences, Nanjing University, Nanjing 210023, China; Co-Innovation Center for Sustainable Forestry in Southern China, Nanjing Forestry University, Nanjing 210037, China; Institute for Plant Molecular Biology, State Key Laboratory of Pharmaceutical Biotechnology, School of Life Sciences, Nanjing University, Nanjing 210023, China; Co-Innovation Center for Sustainable Forestry in Southern China, Nanjing Forestry University, Nanjing 210037, China; Institute for Plant Molecular Biology, State Key Laboratory of Pharmaceutical Biotechnology, School of Life Sciences, Nanjing University, Nanjing 210023, China; Co-Innovation Center for Sustainable Forestry in Southern China, Nanjing Forestry University, Nanjing 210037, China; Institute for Plant Molecular Biology, State Key Laboratory of Pharmaceutical Biotechnology, School of Life Sciences, Nanjing University, Nanjing 210023, China; Co-Innovation Center for Sustainable Forestry in Southern China, Nanjing Forestry University, Nanjing 210037, China; School of Life Sciences and Chemical Engineering, Jiangsu Second Normal University, Nanjing 210013, China; Institute for Plant Molecular Biology, State Key Laboratory of Pharmaceutical Biotechnology, School of Life Sciences, Nanjing University, Nanjing 210023, China; Co-Innovation Center for Sustainable Forestry in Southern China, Nanjing Forestry University, Nanjing 210037, China; Co-Innovation Center for Sustainable Forestry in Southern China, Nanjing Forestry University, Nanjing 210037, China; State Key Laboratory of Crop Genetics and Germplasm Enhancement, Nanjing Agricultural University, Nanjing 210095, China; State Key Laboratory of Crop Genetics and Germplasm Enhancement, Nanjing Agricultural University, Nanjing 210095, China; Institute for Plant Molecular Biology, State Key Laboratory of Pharmaceutical Biotechnology, School of Life Sciences, Nanjing University, Nanjing 210023, China; Institute for Plant Molecular Biology, State Key Laboratory of Pharmaceutical Biotechnology, School of Life Sciences, Nanjing University, Nanjing 210023, China; Institute for Plant Molecular Biology, State Key Laboratory of Pharmaceutical Biotechnology, School of Life Sciences, Nanjing University, Nanjing 210023, China; Co-Innovation Center for Sustainable Forestry in Southern China, Nanjing Forestry University, Nanjing 210037, China; Institute for Plant Molecular Biology, State Key Laboratory of Pharmaceutical Biotechnology, School of Life Sciences, Nanjing University, Nanjing 210023, China; Co-Innovation Center for Sustainable Forestry in Southern China, Nanjing Forestry University, Nanjing 210037, China; Jiangsu Key Laboratory for Eco-Agricultural Biotechnology around Hongze Lake, Huaiyin Normal University, Huai’an 223300, China; Institute for Plant Molecular Biology, State Key Laboratory of Pharmaceutical Biotechnology, School of Life Sciences, Nanjing University, Nanjing 210023, China; Co-Innovation Center for Sustainable Forestry in Southern China, Nanjing Forestry University, Nanjing 210037, China

## Abstract

The low phosphorus (P) availability of acidic soils severely limits leguminous plant growth and productivity. Improving the soil P nutritional status can be achieved by increasing the P-content through P-fertilization or stimulating the mineralization of organic P via arbuscular mycorrhizal fungi (AMF) application; however, their corresponding impacts on plant and soil microbiome still remain to be explored. Here, we examined the effects of AMF-inoculation and P-fertilization on the growth of soybean with different P-efficiencies, as well as the composition of rhizo-microbiome in an acidic soil. The growth of recipient soybean NY-1001, which has a lower P-efficiency, was not significantly enhanced by AMF-inoculation or P-fertilization. However, the plant biomass of higher P-efficiency transgenic soybean PT6 was significantly increased by 46.74%–65.22% through AMF-inoculation. Although there was no discernible difference in plant biomass between PT6 and NY-1001 in the absence of AMF-inoculation and P-fertilization, PT6 had approximately 1.9–2.5 times the plant biomass of NY-1001 after AMF-inoculation. Therefore, the growth advantage of higher P-efficiency soybean was achieved through the assistance of AMF rather than P-fertilization in available P-deficient acidic soil. Most nitrogen (N)-fixing bacteria and some functional genes related to N-fixation were abundant in endospheric layer, as were the P-solubilizing *Pseudomonas plecoglossicida*, and annotated P-metabolism genes. These N-fixing and P-solubilizing bacteria were positive correlated with each other. Lastly, the two most abundant phytopathogenic fungi species accumulated in endospheric layer, they exhibited positive correlations with N-fixing bacteria, but displayed negative interactions with the majority of the other dominant non-pathogenic genera with potential antagonistic activity.

## Introduction

Acidic soils stand for the majority of the world’s potential agricultural land [1], and their characteristics of low pH value, high aluminum (Al) toxicity, and low bioavailable phosphorus (P) directly impact crop roots and inhibit the absorption of water and nutrients, thereby reducing crop growth and yield [2]. Besides being an important source of protein and oil, soybeans are also regarded as one of the pioneers of soil amendments and agricultural production due to their nitrogen (N) fixation capabilities [3]. As a result of the fact that the majority of soybean productions in the world predominantly take place in low-pH areas, many Al-sensitive soybeans as major varieties predominantly used in soybean-producing regions are still limited or suppressed to some extent [4].

Soil microorganisms, the ‘unseen majority’ of terrestrial ecosystem, contribute to the growth, fitness, and health of aboveground plants [5, 6]. Plant growth and development can be enhanced by a wide range of microorganisms, referred to as plant growth promoting rhizobacteria (PGPR) or plant growth promoting fungi (PGPF), due to their capacity in solubilizing phosphates, producing hormones, fixing nitrogen, affecting plant metabolism (e.g., increasing mineral and water uptake), improving root development and increasing enzymatic activity, or suppressing other plants that pathogens dissolve phosphates [7]. Some PGPRs and PGPFs, such as nitrogen fixing bacteria and mycorrhizal fungi are responsible for up to 80% of nitrogen and 75% of phosphorus absorption by plants [8]. For example, rhizobia directly fix nitrogen in the air, while mycorrhiza fungi help plants obtain phosphate indirectly by employing the extensive hyphal network to increase the soil volume that can be explored by plants for phosphate [4, 9]. By promoting plant absorption of nutrients and improving plant resistance to biotic/abiotic stresses, these PGPRs/PGPFs such as rhizobia and arbuscular mycorrhizal fungi (AMF), are important in regulating plant productivity, particularly in low fertility ecosystems [10, 11]. Therefore, there is great agricultural importance to PGPRs/PGPFs in terms of improving soil fertility and crop yield while reducing the negative environmental impact of chemical fertilizers [7, 10].

In the past decades, impressive progress has been achieved in the production, commercialization, and use of microbial inoculants, because of their advantages over agrochemicals on the basis of mitigating environmental impacts and improving yields at low cost [10, 12]. Moreover, with the advancement of omics and bioinformatics technologies, the use of PGPRs and PGPFs as biofertilizers has become an increasingly viable strategy for managing complex rhizosphere interactions in suppressing disease and enhancing production and performance of other plants [7, 13]. Although the biofertilizer fertilizer techniques have been reported to be useful in agricultural production [14], the differential effectiveness and mechanism of their joint applications on soybean with different P-efficiencies in available P-deficient acidic soil need to be fully elucidated and further investigated. Genetic improvement and breeding can generate good economic benefits while combining soil functional microorganisms is expected to become a fast and green new method for soil improvement and crop growth promotion; however, the AMF were found to have impacts on the expressions of phosphate (Pi) transporters (i.e., OsPT1–13) in rice [15], thus the microbial agents may also play a counterproductive role by severely restricting the expression of target genes in transgenic soybean.

**Table 1:** Characteristic and yield of plants.

	**NY-1001**				**PT6**			
**Trait**	**(Mean ± SD)**				**(Mean ± SD)**			
	**CK**	**Ri**	**P**	**Ri + P**	**CK**	**Ri**	**P**	**Ri + P**
Plant dry weight (g)	0.94 ± 0.37	**0.77 ± 0.16**	0.87 ± 0.19	**0.55 ± 0.14**	0.92 ± 0.13^B^	**1.52 ± 0.33** ^**A**^	0.88 ± 0.24^B^	**1.35 ± 0.08** ^**AB**^
100-seed Weight (g)	6.33 ± 2.52	4.76 ± 2.09	5.86 ± 2.48	6.53 ± 0.68	6.64 ± 1.13	6.36 ± 1.17	5.82 ± 1.24	6.27 ± 1.96
C content (%)	41.04 ± 0.2	41.31 ± 1.25	40.99 ± 0.04	43.89 ± 2.29	41.83 ± 0.89	41.94 ± 0.65	40 ± 0.96	41.67 ± 0.48
N content (%)	3.14 ± 0.24	3.83 ± 1.11	2.57 ± 1.65	4.34 ± 1.09	3.31 ± 0.55	3.08 ± 1.35	2.09 ± 0.44	2.88 ± 0.29
C:N ratio	13.12 ± 0.99	11.38 ± 3.10	12.07 ± 2.59	10.55 ± 2.71	12.83 ± 1.75	15.15 ± 5.18	19.63 ± 3.67	14.58 ± 1.46

NY-1001 and PT6 represent the lower P-efficiency recipient soybean ‘NY-1001’ and higher P-efficiency soybean ‘PT6’. CK, Ri, and P represent no treatment, AMF inoculation and phosphorus fertilization, respectively. Plant dry weight and character of seeds were measured at maturity stage (*n* = 3). C and N represent plant carbon and nitrogen contents at maturity stage (*n* = 3). SD stands for standard deviation. The significance test was performed using a one-way ANOVA. The values in bold indicate the significant difference (*P* < 0.05) between the NY-1001 and PT6 groups, while the values without the same superscript letter indicate the significant difference (*P* < 0.05) between treatments within NY-1001 or PT6 groups.

To address these issues, a higher P-efficiency genetically modified (GM) soybean ‘PT6’, and its recipient soybean variety ‘NY-1001’, were used in the present study to assess the alterations in the abundance, composition and function of soil microorganism in response to AMF inoculation together with or without P-fertilization. We aimed to test the following hypotheses: (i) the AMF inoculation and P-fertilization can improve soybean growth and development in acidic soil by altering the rhizosphere microbiome (rhizo-microbiome); (ii) the improvement of phosphate metabolism efficiency of host soybeans can enhance the soybean development in acidic soil, while the effectiveness of this improvement in nutrition and growth depends directly on the ability of soybean to efficiently use phosphorus; and (iii) the selective effect of soybean rhizosphere niche can play a crucial role in improving soybean growth under environmental stress by either enriching beneficial microorganisms that promote plant growth or reducing pathogenic microorganism.

## Results

### Physicochemical properties of soils and plants

AMF inoculation and P-fertilization had no significant effect on the carbon (C) content, nitrogen (N) content or C:N ratio of soybean at maturity (Table 1). Meanwhile, there was no difference in the hundred-grain weight between the AMF inoculation and phosphorus (P) fertilization treatment groups (Table 1). The AMF inoculation and P-fertilization had no significant impact on dry weight of recipient NY-1001; however, the AMF inoculation without/with P-fertilization was found to have a great improvement in dry weight of higher P-efficiency soybean PT6 by 65.22% and 46.74% at maturity (Table 1). Under the condition without AMF inoculation and P-fertilization, the plant biomass of PT6 has no significant difference as compared to NY-1001 (Table 1). Nevertheless, the plant biomass of PT6 was surprisingly found to be much higher than NY-1001 by 97.40% and 145.45% under the AMF inoculation without/with P-fertilization at maturity stage (Table 1). Lastly, the activities of five key enzymes involved in the carbon, N and P cycles in soil did not significantly change between the AMF inoculation and P-fertilization treatment groups (Fig. S1, see online supplementary material).

### Alpha and Beta diversity of root-associated microorganism communities

**Figure 1:**
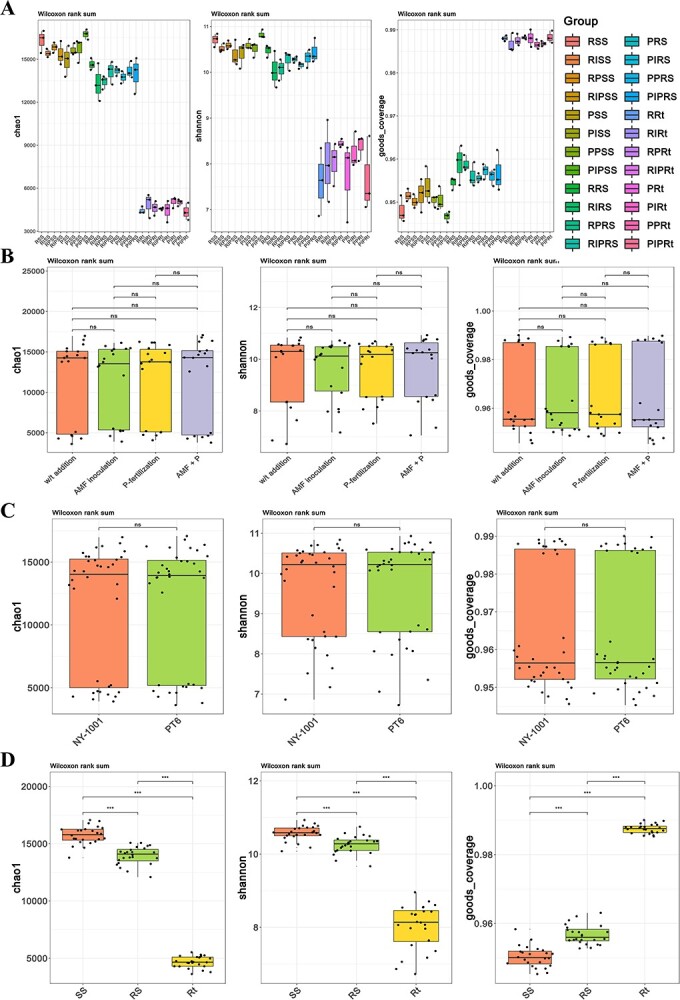
The boxplot of alpha diversity of root-associated bacterial community. **A** The Chao value, Shannon value and Good’s coverage of bacterial community of all groups. The first letter R and P represent the lower P-efficiency recipient soybean ‘NY-1001’ and higher P-efficiency soybean ‘PT6’. I and P in the middle represent AMF inoculation and P-fertilization, respectively. SS, RS, and Rt represent surrounding soils, rhizospheric soils, and intact roots, respectively. The boxplot of combined groups was mapped by using four different AMF inoculation and P-fertilization treatment groups (**B**), two different P-efficiencies of soybean groups (**C**) and three different sampling compartment groups (**D**), respectively. The left, middle and right panels are Chao index, Shannon index and coverage index, respectively. Significance codes: *P* < 0.05; ^**^P < 0.01; and ^***^P < 0.001.

In bacterial and fungal communities, it was found that there was no significant difference in alpha diversity (*P* > 0.05) between different AMF inoculation and P-fertilization treatment groups (Fig. 1A and B; Fig. S2A and B, see online supplementary material). The P-efficiency of soybean also has no impact on alpha diversity (Fig. 1A and C; Fig. S2A and C, see online supplementary material). From SS (surrounding soil) to RS (rhizospheric soil) to Rt (intact roots), the values of the Chao1 and Shannon indices decreased and the coverage index increased (Fig. 1D), which indicate a decline in community diversity and richness while there is an increase in community coverage from the outside to the inside of the root.

**Figure 2:**
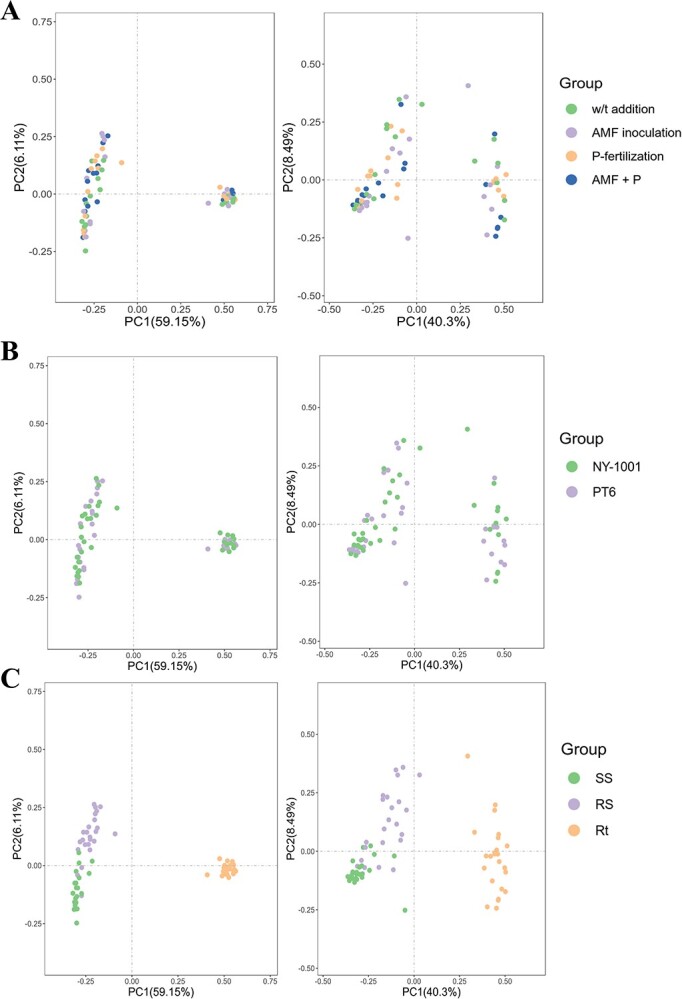
PCoA of microbial communities based on Bray–Curtis distance. **A** PCoA plots for four different AMF inoculation and P-fertilization treatment groups. **B** PCoA plots for two different P-efficiencies of soybean groups. **C** PCoA plots for three different sampling compartment groups. The left panel is PCoA of bacterial community while the right panel is PCoA of fungal community. ‘W/t addition’ and ‘AMF + P’ represent ‘no P-fertilization or AMF inoculation’ and ‘AMF inoculation with P-fertilization’, respectively. NY-1001 and PT6 represent the lower P-efficiency recipient soybean ‘NY-1001’ and higher P-efficiency soybean ‘PT6’. SS, RS, and Rt represent surrounding soils, rhizospheric soils, and intact roots, respectively.

The PCoA and NMDS showed no significant distinction in the distance between the different four AMF inoculation and P-fertilization treatment groups or between two different P-efficiencies of soybean groups in bacterial and fungal communities (Fig. 2A and B; Fig. S3A and B, see online supplementary material). However, there existed a significant difference in distance between niche Rt and the two soil niches (i.e., SS and RS) (Fig. 2C; Fig. S3C, see online supplementary material). The statistical analyses of ANOSIM and Adonis based on the Bray–Curtis distance matrix revealed that there existed no significant difference between different treatment groups, between NY-1001 and PT6 groups or between different host niches groups when the single variable was controlled (Tables S1 and S2, see online supplementary material). Nevertheless, the analysis of the combined statistical data revealed that while no significant distinctions were observed between the treatment groups or the NY-1001 and PT6 groups, substantial variations were identified among the different host niches found in the soybean rhizosphere (Table 2).

**Table 2:** Statistical analyses of root-associated microorganisms communities’ structure.

	**Group vs. Group**	**Adonis**		**ANOSIM**	
		**R** ^**2**^	***P-*value**	**Statistic**	***P*-value**
Bacterial community	Control vs. P-treatment	0.01457	0.726	−0.01351	0.528
	Control vs. RI-treatment	0.01491	0.737	−0.0098	0.461
	Control vs. P + RI-treatment	0.02089	0.398	0.01274	0.235
	P-treatment vs. RI-treatment	0.01536	0.728	−0.01436	0.568
	P-treatment vs. P + RI-treatment	0.01677	0.646	−0.01109	0.478
	RI-treatment vs. P + RI-treatment	0.01953	0.545	0.0057	0.305
	NY-1001 vs. PT6	0.01022	0.491	0.00066	0.321
	SS vs. RS	0.21381	**0.001**	0.5973	**0.001**
	SS vs. Rt	0.6482	**0.001**	1	**0.001**
	RS vs. Rt	0.57816	**0.001**	1	**0.001**

Fungal community	Control vs. P-treatment	0.01481	0.851	−0.03117	0.864
	Control vs. RI-treatment	0.03153	0.268	0.01247	0.256
	Control vs. P + RI-treatment	0.02951	0.341	0.00873	0.279
	P-treatment vs. RI-treatment	0.02474	0.462	−0.0085	0.448
	P-treatment vs. P + RI-treatment	0.02897	0.347	−0.00069	0.355
	RI-treatment vs. P + RI-treatment	0.03336	0.275	0.00786	0.299
	NY-1001 vs. PT6	0.01497	0.305	0.00219	0.295
	SS vs. RS	0.14146	**0.001**	0.32605	**0.001**
	SS vs. Rt	0.4767	**0.001**	0.9818	**0.001**
	RS vs. Rt	0.35162	**0.001**	0.92994	**0.001**

ANOSIM means analysis of similarities and Adonis is used for PERMANOVA (permutational multivariate analysis of variance) based on the Bray–Curtis distance metrics. P and I represent P-fertilization and AMF inoculation, respectively. NY-1001 and PT6 represent the lower P-efficiency recipient soybean ‘NY-1001’ and higher P-efficiency soybean ‘PT6’. SS, RS, and Rt represent surrounding soils, rhizospheric soils, and intact roots, respectively. The *P*-values in bold indicate the significant difference (*P* < 0.05 (^*^)) between groups by the tests.

### Comparison of the compositions of microbial communities **at different taxonomic levels**

The composition and abundance of the host niche Rt were very different from those of SS and RS, according to the histogram of the major phyla of the microbial community (Fig. S4A and B, see online supplementary material). Chloroflexi was the most abundant phylum in niche SS, followed by Proteobacteria, Acidobacteria, and Actinobacteria, while Chloroflexi and Proteobacteria were the two major phyla in niche RS (Table S3, see online supplementary material). However, in niche Rt, Proteobacteria was the most abundant phylum, which occupied around 58–79% of all the bacteria (Table S3, see online supplementary material). Unlike the bacterial community, Ascomycota was the most abundant phylum in fungal community, followed by Basidiomycota, Zygomycota, and Chytridiomycota, which were consistent in all three host niches of the soybean rhizosphere (Table S3, see online supplementary material). The AMF inoculation and P-fertilization treatments had no significant effects on the composition of bacterial community; however, these treatments surprisingly affected the composition of the fungal community at phylum level (Table S3, see online supplementary material). The AMF inoculation with P-fertilization decreased the abundance of Ascomycota in RS and Rt, and also significantly increased the abundance of Basidiomycota in niche Rt for the lower P-efficiency recipient soybean NY-1001 (Table S3, see online supplementary material). Moreover, the AMF inoculation increased the abundance of Zygomycota and Cercozoa in SS for the higher P-efficiency soybean PT6 (Table S3, see online supplementary material).

**Figure 3:**
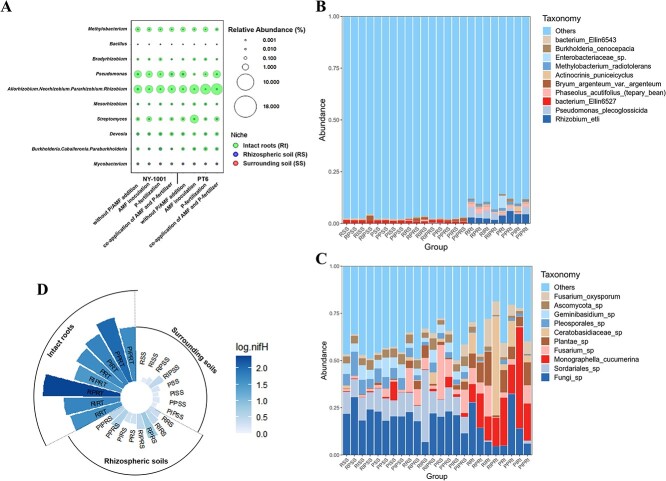
The bubble plot of top 10 nitrogen-fixing bacterial genera (>0.01) and the relative abundances of top 10 species of bacterial and fungal communities. **A** A bubble plot (*n* = 3) displaying the relative abundance of nitrogen-fixing bacteria at the genus level. **B** The relative abundances of top 10 species of bacterial community. **C** The relative abundances of top 10 species of fungal community. **D** Relative abundance of *nifH* gene in soybean root-associated bacterial community; a value of 0 (log_10_nifH) was assigned to the detected value of the rhizospheric soil at two sides of the control lower P-efficiency recipient soybean NY-1001 (RSS). Treatments’ details were as in Fig. 1.

It was also discovered that there was a considerable amount of variation across different host niches when comparing the composition of the microbiome at the genus level (Fig. S4C and D, see online supplementary material). The bacterial genera *Novosphingobium*, *Allorhizobium-Neorhizobium-Pararhizobium-Rhizobium*, *Sphingomonas,* and *Pseudomonas* were enriched in the niche Rt, while the genera *Bryobacter* and *Rhodoplanes* were enriched in the soil niches SS and RS (Fig. S4C, see online supplementary material). In the fungal community, the relative abundance of the genus *Monographella* was higher in the niche Rt, while the abundance of *Geminibasidium* and *Thielavia* was significantly higher in the niche SS (Fig. S4D, see online supplementary material). Further random forest analysis at the genus level showed that *Thermosporothrix*, *Actinospica*, and *Piriformospora* could be used as indicator species (mean decrease Gini >3.0) for grouping four different AMF inoculation and P-fertilization treatments (Fig. S5A and D, see online supplementary material). The genera *Acidothermus*, *Conexibacter*, *Thielavia*, *Gymnopilus*, *Aspergillus*, *Acremonium,* and *Penicillium* were the major genera that potentially contributed to the grouping of three different host niches (Fig. S5B and E, see online supplementary material), while the genera *Devosia* and *Graphostroma* could be used for grouping two different soybeans (Fig. S5C and F, see online supplementary material). Although the AMF inoculation and P-fertilization treatments had no significant effects on the abundance of main identified nitrogen-fixing (N-fixing) bacterial genera (e.g., *Devosia*, *Bradyrhizobium*, *Bacillus*, *Rhizobium,* and *Pseudomonas*) (Fig. 3A; Table S4, see online supplementary material), there existed a significant difference in the relative abundances of some N-fixing bacterial genera in different host niches of the soybean rhizosphere (Table 3). Most of these N-fixing bacterial genera (7/10) with high abundance were enriched in niche Rt as compared to niche RS and SS (e.g., *Devosia*, *Streptomyces*, *Pseudomonas*, and *Bradyrhizobium*) (Table 3 and Fig. 3A). However, the most abundant genus, *Mycobacterium*, and the well-known PGPR genus *Bacillus* had lower relative abundance in niche Rt than in soil niches SS and RS.

**Table 3:** The relative abundance (%) of top 10 nitrogen-fixing bacterial genera from three different host niches.

**Genus**	**SS**	**RS**	**Rt**
*Mycobacterium*	0.493 ± 0.048^a^	0.328 ± 0.032^b^	0.118 ± 0.07^c^
*Burkholderia-Caballeronia-Paraburkholderia*	0.258 ± 0.068^b^	0.368 ± 0.157^b^	0.914 ± 0.431^a^
*Devosia*	0.212 ± 0.059^c^	0.339 ± 0.123^b^	1.176 ± 0.192^a^
*Streptomyces*	0.169 ± 0.029^a^	0.115 ± 0.04^b^	2.701 ± 2.532^ab^
*Mesorhizobium*	0.100 ± 0.029^c^	0.169 ± 0.045^b^	0.562 ± 0.179^a^
*Allorhizobium-Neorhizobium-Pararhizobium-Rhizobium*	0.062 ± 0.031^b^	0.153 ± 0.118^b^	9.796 ± 3.456^a^
*Pseudomonas*	0.061 ± 0.089^b^	0.167 ± 0.138^b^	4.552 ± 2.087^a^
*Bradyrhizobium*	0.055 ± 0.0096^c^	0.0841 ± 0.0229^b^	0.5676 ± 0.2852^a^
*Bacillus*	0.0422 ± 0.008^a^	0.0224 ± 0.0041^b^	0.0059 ± 0.0054^c^
*Methylobacterium*	0.034 ± 0.019^b^	0.073 ± 0.045^b^	2.286 ± 0.594^a^

SS, RS, and Rt represent surrounding soils, rhizospheric soils, and intact roots, respectively. The significance test was performed using a one-way ANOVA. The values with different superscript letters indicate the significant difference (*P* < 0.05) between different host niches of soybean rhizosphere.

At the species level, *Rhizobium etli* and *Pseudomonas plecoglossicida* were the most abundant species in the bacterial community, and their relative abundance was higher in Rt than in SS and RS (Fig. 3B). *Monographella cucumerina* was the most abundant species in the fungal community, followed by *Fusarium oxysporum* and *Mortierella camargensis* (Fig. 3C). The species *M. cucumerina* and *F. oxysporum* were enriched, while *Sordariales sp.* and *Geminibasidium sp.* were depleted in niche Rt (Fig. 3C). Then the quantification of the *nifH* gene was determined by performing quantitative real-time PCR, and the copy number of the *nifH* gene did not differ significantly between different treatments or between different soybeans (Fig. 3D). However, the abundance of the *nifH* gene in Rt was significantly higher than in SS and RS (Fig. 3D), which was consistent with the overall abundance distribution of N-fixing bacteria at the genus and species level.

### Functional analysis of microbial communities

**Figure 4:**
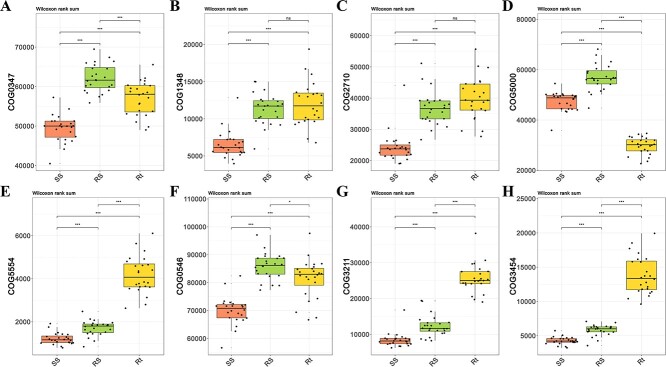
The relative abundance of COG function classification which was directly related to nitrogen-fixation and phosphorus cycle, was mapped by using three different sampling compartment groups. The five nitrogen-fixation COGs from (**A**) to (**E**) were described as nitrogen regulatory protein PII (**A**), nitrogenase subunit NifH (ATPase) (**B**), nitrogenase molybdenum-iron protein, alpha and beta chains (**C**), signal transduction histidine kinase involved in nitrogen fixation and metabolism regulation (**D**), and nitrogen fixation protein (**E**), respectively. The three COGs directly related to soil phosphorus cycle were described as predicted phosphatases (**F**), predicted phosphatase (**G**) and metal-dependent hydrolase involved in phosphonate metabolism (**H**), respectively.

Using the software PICRUSt2 and FUNGuild to compare the composition and abundance of functional genes in root-associated microbial communities, no significant differences were found between different treatments (Fig. S6A and B, see online supplementary material). Then, a total of five clusters of orthologous groups (COG) function classifications were discovered to be directly related to N-fixation: COG0347, COG1348, COG2710, COG5000, and COG5554, which were also found to have no significant difference between different AMF inoculation and P-fertilization treatments (Fig. S6C–J, see online supplementary material). The relative abundances of COG0347, COG1348, and COG2710 were higher in niches Rt and RS than in SS, while the abundance of COG5554, also known as N fixation protein, was significantly higher in Rt than in RS and SS (Fig. 4). However, COG5000, characterized as a signal transduction histidine kinase involved in N fixation and metabolism regulation, was depleted in soil niche Rt as compared to SS and RS (Fig. 4D). Three COG function classifications were also discovered to be directly related to P-cycle. Results revealed that these three COGs exhibited no significant difference following AMF inoculation and P-fertilization (Fig. S6F–H). Among them, the relative abundance of COG3211 and COG3454 (described as predicted phosphatase and metal-dependent hydrolase involved in phosphonate metabolism, respectively) was significantly higher in Rt compared to RS and SS (Fig. 4G and H).

### Co-occurrence patterns among microbes residing soybean rhizosphere

To understand the feedbacks that occur among soybean rhizosphere soils, the co-occurrence patterns of bacterial and fungal communities were analysed (Fig. S7, see online supplementary material). Pearson-correlation analysis revealed a significant positive correlation (*r* > 0, *P* < 0.05) between the N-fixing bacterial genera. For example, the high abundance bacterium *Methylobacterium* exhibited co-occurrence correlations with *Pseudomonas* (*r* = 0.460, *P* = 0.00004), *Streptomyces* (*r* = 0.351, *P* = 0.0025), *Devosia* (*r* = 0.786, *P* = 2 × 10^−16^), *Allorhizobium*-*Neorhizobium*-*Pararhizobium*-*Rhizobium* (*r* = 0.716, *P* = 2 × 10^−12^) and *Burkholderia*-*Caballeronia*-*Paraburkholderia* (*r* = 0.487, *P* = 0.00001) (Fig. S7A, see online supplementary material). In the fungal community, the well-known phytopathogenic and toxigenic fungi *Fusarium* have positive co-occurrence correlations with *Arthrobotrys* (*r* = 0.354, *P* = 0.0023) and *Strelitziana* (*r* = 0.243, *P* = 0.0398), whereas it showed negative interactions with the rest of the genera (Fig. S7B, see online supplementary material). Another pathogen, *Monographella*, was also only found to have positive correlations with *Graphostroma* (*r* = 0.384, *P* = 0.0009), *Hannaella* (*r* = 0.322, *P* = 0.0058) and *Cyphellophora* (*r* = 0.262, *P* = 0.0260). The indicator PGPF species *Piriformospora*, was negatively correlated with all other genera except *Acremonium* (*r* = 0.774, *P* = 2 × 10^−15^) (Fig. S7B, see online supplementary material). Fig. S7C (see online supplementary material) showed the analysis results of the correlation patterns between bacteria and fungi, in which *Fusarium* has positive co-occurrence correlations with *Novosphingobium* (*r* = 0.256, *P* = 0.0303), and *Monographella* was found to have significant positive correlations with *Novosphingobium* (*r* = 0.587, *P* = 6 × 10^−8^), *Allorhizobium*-*Neorhizobium*-*Pararhizobium*-*Rhizobium* (*r* = 0.655, *P* = 4 × 10^−10^), *Sphingomonas* (*r* = 0.409, *P* = 0.0003), *Streptomyces* (*r* = 0.386, *P* = 0.0007) and *Afipia* (*r* = 0.376, *P* = 0.0012).

## Discussion

### The effects of the AMF inoculation on plant were host-P-efficiency-dependent

To eliminate the possible uncontrollable effects of genotypic differences in host soybean on the root-associated microbial communities, the lower P-efficiency recipient soybean ‘NY-1001’ and higher P-efficiency transgenic soybean ‘PT6’ with same genetic background were used as plant materials in this study. Our results showed that AMF inoculation, as well as P-fertilization, affected some traits of plants, significantly improved the dry weight of higher P-efficiency soybean PT6 in particular (Table 1), which confirmed previous research that mycorrhizal inoculation and increasing levels of P application had a positive effect on plant shoot dry weight [16]. Moreover, compared with lower P-efficiency NY-1001, AMF inoculation and P-fertilization treatments had better improvement effects on higher P-efficiency soybean PT6 by increasing its dry weight at maturity by 65.22% and 46.74% in an available P-deficient environment (Table 1). *OsPT6* is predominately expressed in the roots and leaves in response to Pi starvation, and the transgenic plants PT6 (overexpression OsPT6) grew better and exhibited significant increases in plant height, root length, root weight, number of pods and seeds, and seed weight per plant compared with the receptor plants under low-Pi of standard Hoagland’s nutrient solution [39]. However, in the present study, the plant biomass of PT6 had no significant difference as compared to NY-1001 under the condition without AMF inoculation and P-fertilization, while the plant biomass of PT6 was much higher than NY-1001 by 97.40% and 145.45% under the AMF inoculation without or with P-fertilization at maturity stage (Table 1). Thus, there might be multiple factors that limit the growth and development of soybean in acidic soils beside the lack of available phosphorus, such as the soil acidity, aluminum toxicity, or N-limitation. Focusing on changes in the composition and function of rhizosphere microorganisms, the AMF inoculation with P-fertilization were found to increase the abundance of Zygomycota and Cercozoa in PT6, while the abundance of Zygomycota in PT6 was also higher than NY-1001 under the AMF inoculation with P-fertilization. These protists together with fungi acted as central nodes in soil biota aggregate networks, took part in soil structuring and C cycling, and contributed significantly to the overall ecosystem [17, 18]. In addition, although there existed no significant difference, the AMF inoculation without P-fertilization increased the soil sucrase enzyme activity by 32.84% as compared to no AMF inoculation and P-fertilization treatment in the PT6 group (Fig. S1, see online supplementary material). Thus, the enrichment of fungi together with protists, which are closely related to stabilization of soil organic carbon might accelerate carbon cycle of soil microenvironment, and ultimately lead to the promotion of plant biomass of soybean PT6 under AMF inoculation. As shown in Fig. 3A, the AMF inoculation also increased the abundance of *Streptomyces* in intact roots (Rt). *Streptomyces* is known as the largest antibiotic-producing genus, which produces a wide variety of bioactive compounds such as antibacterial, antifungal, and antiparasitic drugs, responsible for the production of over two-thirds of the clinically useful antibiotics of natural origin [19]. That is to say, AMF inoculation also participated in helping soybeans PT6 gain growth advantages by recruiting *Streptomyces* in RT. In a word, AMF inoculation was found to improve the tolerance of soybean to acidic soil mainly by recruiting specific PGPRs and altering the root-associated microbiome assembly in a host-dependent manner in this study [20]. Although it is not ruled out that other genetic breeding methods may directly enable plants to gain growth advantages in acidic soil, this experiment reminds us that the effectiveness of introducing exogenous excellent genes through genetic breeding still needs to be verified in actual agricultural situations. In addition, we also found that the shoot biomass of both lower and higher P-efficiency soybean decreased after the application of P-fertilizer (e.g., P vs. CK and Ri + P vs. Ri) (Table 1), even if there existed no significant difference between P-fertilization and non-P-fertilization groups. A previous study showed that the application of P-fertilizer reduces the colonization of AMF [21], which might lead to a reduction in soybean biomass. These results revealed that, in addition to improving crop varieties, optimization of supporting cultivation methods is also important. Although the expressions of transformed Pi transporter gene might be suppressed by AMF [15], we need to consider the combined use of suitable AMF agents rather than single chemical P-fertilization when verifying the growth advantage of higher P-efficiency soybean in the available P-deficient acidic soil.

### Host niches determined the differentiation of microbiome assembly

Our results are also consistent with previous findings, that the host niches of soybean (i.e., sampling compartment) determined the differentiation of the assembly and the shift of rhizo-microbiome [22]. Each sampling compartment contained different potential niches for microbes, primarily determined by its physical structure, biotic components, and abiotic conditions [23]. In this study, some representative PGPRs such as *Sphingomonas* and *Pseudomonas* were significantly enriched in Rt (Fig. S4C, see online supplementary material). These rhizobacteria can promote plant growth by improving nutrient absorption and reducing disease levels, potentially increasing crop grain yield in low-N and low-P soils [24]. Most N-fixing bacterial genera, *R. etli* (one of the two most abundant N-fixing species), the *nifH* gene, and the N-fixation COG function classifications genes were found to be enriched in the niche Rt (Table 3 and Fig. 4; Fig. S4E, see online supplementary material). According to these results, the soybean root enriched N-fixing functional genes by enriching the aforementioned N-fixing genera and species, which is consistent with our previous researches [20, 25]. *P. plecoglossicida*, the second most abundant bacteria at the species level, and metal-dependent hydrolase involved in phosphonate metabolism COG3454, both showed higher relative abundance in Rt than in SS and RS (Figs 3B and 4H). As a well-known phosphate-solubilizing bacteria (PSB), *P. plecoglossicida* improved crop growth and grain yield, promoted plant P uptake and participated in controlling plant disease by increasing enzyme activities and promoting the content of available P [26]. Thus, the enrichment effect of the functional bacteria closely related to N-fixing and P-solubilizing in the soybean root was beneficial to soybean nutrient absorption and growth in the acidic soils with low P-availability.

In contrast to the trend mentioned above, the rhizosphere of soybean (i.e., soil niches SS and RS) was found to have positive selections (i.e., enrichment effects) for the well-known PGPR genus *Bacillus* [27] and the most abundant N-fixing genus *Mycobacterium* as compared to niche Rt [28] (Table 3). The host selection pressure increases successively from rhizospheric soil to the endospheric layer in the soil–plant continuum, and root-secreted organic acids can attract specific beneficial microbes, resulting in the differentiation of the assembly and shift of rhizo-microbiome [29]. A previous study reported that *Bacillus* and *Mycobacterium* in nutrient-deficient soil promoted plant growth and nutrient (N, P, and K) uptake by crops [30]. Consequently, it might be hypothesized that the soil niches also played a role in providing a suitable living environment for specific PGPRs, which led to better host soybean growth in acidic soil.

### Microbial tools have broad application prospects in ecological agriculture

P-fertilization can directly improve soil phosphorus nutritional status, organic phosphorus can be mineralized, and root phosphatase activity can be enhanced by AMF application [31]. Additionally, the changes in P-nutrition caused by AMF inoculation and P-fertilization may have an impact on other important enzyme activities [32]. Thus, we initially anticipated that AMF inoculation and P-fertilization would improve soybean tolerance by altering the rhizosphere microbial communities in the present study. However, the activities of five key enzymes related to the C, N, and P cycles in soil revealed no difference between AMF inoculation and P-fertilization treatment groups (Fig. S1, see online supplementary material). The exact causes of this finding are unknown, which may be related to the sampling stages, genotype and P-efficiency of the host plant. In this study, only the AMF inoculation and P-fertilization treatments were found to have significant influences on the assembly and shift of the rhizo-microbiome at different taxonomy level (Fig. 3; Table S3, see online supplementary material ). The improvement of soybean growth and development such as shoot biomass was directly related to the increase in colonization ability of some specific PGPRs and PGPFs caused by the AMF inoculation. Thus, the application of crops with excellent traits (such as some transgenic crops) in ecological agriculture might require the assistance of specific and matched cultivation methods such as appropriate microbial agents. When analysing the correlation patterns within and between bacteria and fungi, the results of Pearson correlation analysis showed a significant positive correlation among the N-fixing bacterial genera (Fig. S7A, see online supplementary material), while the pathogenic fungi *Fusarium* and *Monographella* which accumulated in Rt have unique positively correlated or negatively correlated fungal genera (Fig. S7B, see online supplementary material) [33, 34]. These pathogenic fungi were also surprisingly found to have significant positive correlations with some N-fixing bacteria such as *Novosphingobium* (Fig. S7C, see online supplementary material). These bacteria and fungi with significant positive and negative correlations can be used for dynamic monitoring of microbial communities. To sum up, this study provides a theoretical basis for the evaluation of the effects of the application of microbial agents in ecological agriculture, enhancement of the effectiveness of special microbial agents, antagonism against pathogenic microorganisms, and the early detection and prevention of pathogens in the future.

AMF inoculation had no effect on plant dry weight of NY-1001 (Table 1). In complex rhizosphere microbial communities, adding AMF may affect the entire rhizosphere microbiota, alter the rhizosphere microecology, and ultimately affect the growth and development of the host plant through the microbe-microbe and microbe-plant interactions, which were closely related to the root exudates and *in situ* microbial composition of host plant [25]. Thus, it is also suggested that plant breeding should consider the complicated interactions between rhizo-microbiome and host soybean with different P-efficiencies in addition to AMF inoculation in available P-deficient acidic soils. One significant limitation of our study was that only vegetative traits were evaluated. Future studies should examine whether and how the effects of P-efficiency of soybean, AMF inoculation, and P-fertilization affect vegetative traits, the expression of the Pi transporter gene, the availability of P content in the soil and the soybean yield traits in stressful environments. As a result of global climate change, land degradation, and the loss of biodiversity, traditional synthetic chemical fertilizer technologies were stretched thin [35]. Although the application of agricultural microbiome is still limited by low efficacy and inconsistent field performance, by harnessing microbiomes in ecological fertilizer to renovate ecological fertilizer technology, such as the development of synthetic microbial communities and novel prebiotics, we are expected to improve plant productivity and abolish hunger [35, 36]. The soil microbiome offers a range of ecosystem services to plants (e.g., nutrient acquisition and tolerance to environmental stress), but some of the basic molecular mechanisms in this field, such as the interaction between host-microbe microbe-microbe, require extensive exploration [37, 38].

## Conclusion

In summary, our results revealed that the AMF inoculation altered the colonization of specific PGPFs and pathogens, and increased the soybean growth, while these effects were directly correlated to the P-metabolism efficiency of the host soybean. The potential growth advantage of higher P-efficiency transgenic soybean PT6 was released only after applying AMF. Thus, the application of crops with excellent traits in ecological agriculture requires the assistance of specific and matched cultivation methods such as appropriate AMF inoculation rather than single P-fertilization, to achieve growth advantage in available P-deficient acidic soil. The soybean root also had a selective effect by enriching nutrient uptake functional PGPRs/PGPFs, which have positive correlations in endospheric layer, to help soybeans obtain better growth and development in acidic soil with low P-availability. Lastly, the plant pathogens exhibited unique positive microbiota and displayed negative interactions with the majority of other dominant non-pathogenic genera with potential antagonistic activity. The present study is expected to explore the growth-promoting efficiency, alone with the assembly mechanism of rhizo-microbiome of different P-efficiencies soybeans in acidic soil under AMF inoculation and P-fertilization, to supply a scientific basis for the selection and application of possible PGPRs/PGPFs agents outside of traditional synthetic chemical fertilizer technology, and to provide data with reference value in breeding and ecological agriculture.

## Materials and methods

### Materials and sampling methods

In this study, a recipient soybean variety ‘NY-1001’ as a control and its transgenic soybean ‘PT6’ (produced by the insertion of the high-affinity phosphate transporter gene *OsPT6*) were selected as two soybean materials with different P-efficiencies. The *OsPT6* gene (Genebank ID: AF536966) coding region introduced the *SacI* and *XbaI* restriction sites by PCR and then inserted into the vector pCAMBIA3301. The plant expression vector was successfully constructed with a *bar* gene as a selection gene and an intron-*gus* reporter gene encoding β-glucuronidase. *OsPT6* was driven by the constitutive cauliflower mosaic virus (CaMV) 35S promoter in the binary vector pCAMBIA3301-OsPT6. *Agrobacterium tumefaciens* strain EHA105 was used for transformation, and the stable heritability of soybean was tested by using Southern blot, Basta painting, GUS assay, and PCR analysis. The overexpression of the *OsPT6* gene was detected by quantitative RT-PCR analysis [39]. The experiment was conducted at Nanjing University, Nanjing, China (32.125 N, 118.965 E). The national microbial resources platform number of *Rhizophagus intraradices* (*RI*) is 1511C0001BGCAM0062, which was provided by the Institute of Plant Nutrition and Resources, Beijing Academy of Agriculture and Forestry Sciences (Beijing, China). The acidic red soil had a pH of 4.54 (± 0.21) and a water holding capacity of 29.3%, which was collected from the Ecological Experiment Station of Red Soil in Yingtan, Jiangxi Province, China (28.208 N, 116.937 E) [25].

A total of 10 g of AMF inoculum was added to about 2 cm below the surface of the soil in each rhizobox before planting. The basic parameters of rhizobox (patent number CN102175487) were descripted in a previous study [40] with minor modification (Fig. S8, see online supplementary material). The length*width*height of the rhizobox is 20 cm*15 cm*20 cm and approximately 3.6 kg of acidic soil was placed in each rhizobox. Two layers of nylon mesh are used to separate every compartment to prevent roots from growing into the compartments on both sides. After being disinfected with chlorine for 16 h, the soybean seeds were washed with sterile water four times, followed by disinfection with 70% ethanol for 30 s and with 2.5% sodium hypochlorite for 5 min, and finally washed with sterile water again four times. Soybeans were planted in the central compartment of the rhizobox. Then the P-fertilization was sprayed (100 mg/kg soil) three times and only used for P fertilization treatment on 18 August, 25 August and 1 September, respectively [41]. Other less important details were listed in our previous studies [25, 40]. The experimental plant and soil were collected at flowering stage (R2 stage, 5 November 2020). Two plants were collected in each rhizobox, and the C and N content of plant was measured in the Center of Modern Analysis at Nanjing University by using the Perkin-Elmer 240c analyzer [25].

The roots and soil were collected carefully to keep away from the air-soil interface to avoid false environments [42]. We divided the root microorganisms into three layers according to the distance between the soil and the root. From outside to inside of the roots, there are surrounding soil (SS), rhizospheric soil (RS), and endospheric layer (Rt), respectively. The SS samples represent the soil in the compartments on both sides of the rhizobox, which is the farthest host niche from the root (Fig. S8, see online supplementary material) [40]. RS was the soil that tightly adhered to the root surface and was collected by brushing with phosphate buffered saline (PBS), while another host niche, the Rt, which represents a mixed sample of the soybean rhizoplane and endosphere, was obtained by further washing the root with PBS twice after collecting RS, followed by centrifuging at 4000 *g*, washing with PBS, and grinding with liquid nitrogen [25, 43]. Part of the rhizospheric soil samples (RS) were pre-treated by being air-dried and filtering through a mesh sieve (50 μm), followed by measuring the five key enzymes involved in the carbon, N and P cycles in soil via corresponding kits purchased from Solarbio Science & Technology Co., Ltd (Beijing, China), their enzyme commission numbers are EC 1.7.99.3 (nitrite reductase, S-NiR), EC 3.5.1.5 (urease, S-UE), EC 3.2.1.26 (sucrase, S-SC), EC 3.1.3.2 (acid phosphatase, S-ACP), and EC 3.1.3.1 (alkaline phosphatase, S-AKP/ALP), respectively [25].

### DNA extraction**,** DNA amplicon sequencing and analyses

The PowerSoil DNA Isolation Kit (MoBio Laboratories Inc., Carlsbad, CA, USA) was used to isolate metagenomic DNA from approximately 0.5 g of SS, RS, and Rt samples, and the detailed methods have been published previously [25]. Following that, the DNA samples were evaluated with a 1% agarose gel, and their concentration was verified with a Qubit Fluorometer to ensure they were more than 10 ng/μl (Invitrogen, Carlsbad, USA) [44].

The forward primer 338F (5’-ACTCCTACGGGAGGCAGCAG-3′) and reverse primer 806R (5’-GGACTACHVGGGTWTCTAAT-3′) were used for the sequencing of V3-V4 hypervariable regions of the 16S rRNA (approximately 468 bp) [45], while the forward primer ITS1F (5’-CTTGGTCATTTAGAGGAAGTAA-3′) and reverse primer ITS2R (5’-GCTGCGTTCTTCATCGATGC-3′) were used for the sequencing of the ITS1 region of the internal transcribed spacer (approximately 350 bp) [46]. The DNA amplicon sequencing via the Illumina MiSeq platform (Illumina, CA, USA) was performed by OE Biotech. Co., Ltd (Shanghai, China) using the MiSeq Reagent Kit. The accession numbers (BioProject ID) of the total of 144 sequencing clean data sets (72 16S rRNA and 72 ITS) associated with this study in the Sequence Read Archive (SRA) are PRJNA884153 and PRJNA884158. Their reviewer links were listed in ‘Data availability statement’.

Data from 16S rRNA amplicon sequencing was analysed using the database silva132/16 s (http://www.arb-silva.de), while data from ITS amplicon sequencing was analysed using Unite7.1/ITS_fungi (http://unite.ut.ee/index.php). An analysis of alpha diversity was conducted using Chao1 (an indicator as community richness), Shannon (as community diversity), and Coverage (as community coverage) indices, and principal co-ordinates analysis (PCoA) and non-metric multidimensional scaling analysis (NMDS) were used for the analysis of beta diversity [47, 48]. The software programs PICRUSt and FUNGuild were used to analyse functional gene compositions and abundances of the rhizo-microbiome. The bioinformatic analyses above were performed via the OECloud platform (https://cloud.oebiotech.cn).

### Quantification of *nifH* by quantitative real-time PCR

In order to determine the abundances of the *nifH* gene, a quantitative real-time PCR (qPCR) assay was performed using primer pairs 338F/518R as an internal control, and PolF/PolR as a quantification primer pair [49]. About 50 ng DNA, 1 μL primer pairs (5 pM), and 10 μL of 2 × SYBR Green mixture (Roche, Switzerland) were present in the final reaction volume of 20 μL. Each DNA sample had three technical duplicates and was incubated for 10 minutes at 95°C, followed by 40 cycles of 15 seconds at 95°C and 1 minute at 60°C [25].

### Statistical analyses

Based on the Bray–Curtis distance metric, the software R (v3.1.3) was used to perform the analysis of similarities (ANOSIM), as well as PERMANOVA analysis (Adonis) [50]. By using the software GraphPad Prism (Version 8.0), the significance of the differences in the data was determined by using one-way ANOVA followed by the Tukey test (*P < 0.05) [25].

## Acknowledgments

This work was supported by grants from the National Natural Science Foundation of China (32101383, 42377327), the China Postdoctoral Science Foundation (2023 T160299, 2022 M711562, 2023 M731604), the Natural Science Foundation of Jiangsu Bureau of Science and Technology (BK20230787), and the Program for Changjiang Scholars and Innovative Research Team in University from the Ministry of Education of China (IRT_14R27).

## Author contributions

Conceptualization and methodology: Y.Y., Z.W., and G.L.; validation and investigation: Z.W., M.Y., A.F., H.H., and H.L.; material supply: Y.Y., Y.Z., and S.Y.; software, data curation and formal analysis: Z.W., M.Y., A.F., H.H., H.L., T.Y., Y.Z., S.Y., K.N., S.S., and J.Q.; writing and visualization: Y.Y., G.L., Z.W., M.Y., A.F., and H.H.; supervision, project administration and funding acquisition: Y.Y., Z.W., and M.Y. All authors read and approved the final manuscript.

## Data availability statement

The accession numbers of all clean sequencing data are PRJNA884153 (https://dataview.ncbi.nlm.nih.gov/object/PRJNA884153?reviewer=ub3bpmjrfu97gbdc173vjc7ev6) and PRJNA884158 (https://dataview.ncbi.nlm.nih.gov/object/PRJNA884158?reviewer = 7pfttkrp8cpbnpi1ssguot7gho).

## Conflict of interests 

This study did not involve endangered species. The authors declared no conflicts of interest.

## Supplementary information

Supplementary data is available at *Horticulture Research* online.

## Supplementary Material

Web_Material_uhae067

## References

[1] Vonuexkull HR , Mutert E. Global extent, development and economic-impact of acid soils. Plant Soil. 1995;171:1–15

[2] Zhang S , Zhou J, Wang G. et al. The role of mycorrhizal symbiosis in aluminum and phosphorus interactions in relation to aluminum tolerance in soybean. Appl Microbiol Biotechnol. 2015;99:10225–35 26278539 10.1007/s00253-015-6913-6

[3] Cheng FX , Cao GQ, Wang XR. et al. Isolation and application of effective nitrogen fixation rhizobial strains on low-phosphorus acid soils in South China. Chin Sci Bull. 2009;54:412–20

[4] Lin MH , Gresshoff PM, Ferguson BJ. Systemic regulation of soybean nodulation by acidic growth conditions. Plant Physiol. 2012;160:2028–39 23054568 10.1104/pp.112.204149 PMC3510129

[5] Zhang RF , Vivanco JM, Shen QR. The unseen rhizosphere root-soil-microbe interactions for crop production. Curr Opin Microbiol. 2017;37:8–14 28433932 10.1016/j.mib.2017.03.008

[6] Miki T , Ushio M, Fukui S. et al. Functional diversity of microbial decomposers facilitates plant coexistence in a plant-microbe-soil feedback model. Proc Natl Acad Sci USA. 2010;107:14251–6 20663953 10.1073/pnas.0914281107 PMC2922608

[7] Perez-Montano F , Alias-Villegas C, Bellogin RA. et al. Plant growth promotion in cereal and leguminous agricultural important plants: from microorganism capacities to crop production. Microbiol Res. 2014;169:325–36 24144612 10.1016/j.micres.2013.09.011

[8] Xie XG , Zhang FM, Yang T. et al. Endophytic fungus drives nodulation and N_2_ fixation attributable to specific root exudates. MBio. 2019;10:e00728–19 31311876 10.1128/mBio.00728-19 PMC6635524

[9] Evelin H , Kapoor R, Giri B. Arbuscular mycorrhizal fungi in alleviation of salt stress: a review. Ann Bot. 2009;104:1263–80 19815570 10.1093/aob/mcp251 PMC2778396

[10] Paries M , Gutjahr C. The good, the bad, and the phosphate: regulation of beneficial and detrimental plant-microbe interactions by the plant phosphate status. New Phytol. 2023;239:29–46 37145847 10.1111/nph.18933

[11] Grumberg BC , Urcelay C, Shroeder MA. et al. The role of inoculum identity in drought stress mitigation by arbuscular mycorrhizal fungi in soybean. Biol Fert Soils. 2015;51:1–10

[12] Santos MS , Nogueira MA, Hungria M. Microbial inoculants: reviewing the past, discussing the present and previewing an outstanding future for the use of beneficial bacteria in agriculture. AMB Express. 2019;9:205 31865554 10.1186/s13568-019-0932-0 PMC6925611

[13] Bashan Y , de-Bashan LE, Prabhu SR. et al. Advances in plant growth-promoting bacterial inoculant technology: formulations and practical perspectives (1998-2013). Plant Soil. 2014;378:1–33

[14] Adnan M , Fahad S, Zamin M. et al. Coupling phosphate-solubilizing bacteria with phosphorus supplements improve maize phosphorus acquisition and growth under lime induced salinity stress. Plants (Basel). 2020;9:900 32708749 10.3390/plants9070900 PMC7411598

[15] Chen XW , Wu FY, Li H. et al. Phosphate transporters expression in rice (*Oryza sativa* L.) associated witharbuscular mycorrhizal fungi (AMF) colonization under different levels ofarsenate stress. Environ Exp Bot. 2013;87:92–9

[16] Almagrabi OA , Abdelmoneim TS. Using of arbuscular mycorrhizal fungi to reduce the deficiency effect of phosphorous fertilization on maize plants (*Zea mays* L.). Life Sci J. 2012;9:1648–54

[17] Fan W , Wu JG. Changes in soil fungal community on SOC and POM accumulation under different straw return modes in dryland farming. Ecosyst Health Sustain. 2021;7:1935326

[18] Pellegrino E , Piazza G, Helgason T. et al. Eukaryotes in soil aggregates across conservation managements: Major roles of protists, fungi and taxa linkages in soil structuring and C stock. Soil Biol Biochem. 2021;163:108463

[19] Chater KF , Biro S, Lee KJ. et al. The complex extracellular biology of *Streptomyces*. FEMS Microbiol Rev. 2010;34:171–98 20088961 10.1111/j.1574-6976.2009.00206.x

[20] Wen ZL , Yang MK, Du MH. et al. Enrichments/Derichments of root-associated bacteria related to plant growth and nutrition caused by the growth of an *EPSPS*-transgenic maize line in the field. Front Microbiol. 2019;10:1335 31275269 10.3389/fmicb.2019.01335 PMC6591461

[21] Nahar K , Bovill B, McDonald G. Mycorrhizal colonization in bread wheat varieties differing in their response to phosphorus. J Plant Nutr. 2020;44:29–45

[22] Anzalone A , Di Guardo M, Bella P. et al. Bioprospecting of beneficial bacteria traits associated with tomato root in greenhouse environment reveals that sampling sites impact more than the root compartment. Front Plant Sci. 2021;12:637582 33927735 10.3389/fpls.2021.637582 PMC8078776

[23] Aas AB , Andrew CJ, Blaalid R. et al. Fine-scale diversity patterns in belowground microbial communities are consistent across kingdoms. FEMS Microbiol Ecol. 2019;95:fiz058 31049552 10.1093/femsec/fiz058

[24] Chen L , Li KK, Shang JY. et al. Plant growth-promoting bacteria improve maize growth through reshaping the rhizobacterial community in low-nitrogen and low-phosphorus soil. Biol Fertil Soils. 2021;57:1075–88

[25] Wen ZL , Yang MK, Han HW. et al. Mycorrhizae enhance soybean plant growth and aluminum stress tolerance by shaping the microbiome assembly in an acidic soil. Microbiol Spectr. 2023;11:e03310–22 36916950 10.1128/spectrum.03310-22 PMC10100836

[26] Kaur G , Reddy MS. Effects of phosphate-solubilizing bacteria, rock phosphate and chemical fertilizers on maize-wheat cropping cycle and economics. Pedosphere. 2015;25:428–37

[27] Ahmad F , Ahmad I, Khan MS. Screening of free-living rhizospheric bacteria for their multiple plant growth promoting activities. Microbiol Res. 2008;163:173–81 16735107 10.1016/j.micres.2006.04.001

[28] Sellstedt A , Richau KH. Aspects of nitrogen-fixing *Actinobacteria*, in particular free-living and symbiotic *Frankia*. FEMS Microbiol Lett. 2013;342:179–86 23461635 10.1111/1574-6968.12116

[29] Xiong C , Zhu YG, Wang JT. et al. Host selection shapes crop microbiome assembly and network complexity. New Phytol. 2021;229:1091–104 32852792 10.1111/nph.16890

[30] Egamberdiyeva D . The effect of plant growth promoting bacteria on growth and nutrient uptake of maize in two different soils. Appl Soil Ecol. 2007;36:184–9

[31] Ortiz N , Armada E, Duque E. et al. Contribution of arbuscular mycorrhizal fungi and/or bacteria to enhancing plant drought tolerance under natural soil conditions: effectiveness of autochthonous or allochthonous strains. J Plant Physiol. 2015;174:87–96 25462971 10.1016/j.jplph.2014.08.019

[32] Ferreira PAA , Tiecher T, Tiecher TL. et al. Effects of *Rhizophagus clarus* and P availability in the tolerance and physiological response of *Mucuna cinereum* to copper. Plant Physiol Biochem. 2018;122:46–56 29175636 10.1016/j.plaphy.2017.11.006

[33] VanWees SCM , Pieterse CMJ, Trijssenaar A. et al. Differential induction of systemic resistance in *Arabidopsis* by biocontrol bacteria. Mol Plant-Microbe Interact. 1997;10:716–24 9245833 10.1094/MPMI.1997.10.6.716

[34] Ma LJ , van der Does HC, Borkovich KA. et al. Comparative genomics reveals mobile pathogenicity chromosomes in *Fusarium*. Nature. 2010;464:367–73 20237561 10.1038/nature08850 PMC3048781

[35] Hu HW , Chen QL, He JZ. The end of hunger: fertilizers, microbes and plant productivity. Microb Biotechnol. 2022;15:1050–4 34767687 10.1111/1751-7915.13973 PMC8966006

[36] Batista BD , Singh BK. Realities and hopes in the application of microbial tools in agriculture. Microb Biotechnol. 2021;14:1258–68 34156754 10.1111/1751-7915.13866 PMC8313292

[37] van der Voort M , Kempenaar M, van Driel M. et al. Impact of soil heat on reassembly of bacterial communities in the rhizosphere microbiome and plant disease suppression. Ecol Lett. 2016;19:375–82 26833547 10.1111/ele.12567

[38] Vorholt JA , Vogel C, Carlstrom CI. et al. Establishing causality: Opportunities of synthetic communities for plant microbiome research. Cell Host Microbe. 2017;22:142–55 28799900 10.1016/j.chom.2017.07.004

[39] Yan W , Chen GH, Yang LF. et al. Overexpression of the rice phosphate transporter gene enhances tolerance to low phosphorus stress in vegetable soybean. Sci Hortic. 2014;177:71–6

[40] Li YC , Li YF, Yang MK. et al. Changes of microbial functional capacities in the rhizosphere contribute to aluminum tolerance by genotype-specific soybeans in acid soils. Biol Fert Soils. 2020;56:771–83

[41] Sun DS , Bi QF, Li KJ. et al. Significance of temperature and water availability for soil phosphorus transformation and microbial community composition as affected by fertilizer sources. Biol Fert Soils. 2018;54:229–41

[42] Peiffer JA , Spor A, Koren O. et al. Diversity and heritability of thez maize rhizosphere microbiome under field conditions. Proc Natl Acad Sci USA. 2013;110:6548–53 23576752 10.1073/pnas.1302837110 PMC3631645

[43] Inceoglu O , Salles JF, van Overbeek L. et al. Effects of plant genotype and growth stage on the betaproteobacterial communities associated with different potato cultivars in two fields. Appl Environ Microbiol. 2010;76:3675–84 20363788 10.1128/AEM.00040-10 PMC2876460

[44] Kennedy K , Hall MW, Lynch MDJ. et al. Evaluating bias of Illumina-based bacterial 16S rRNA gene profiles. Appl Environ Microbiol. 2014;80:5717–22 25002428 10.1128/AEM.01451-14 PMC4178620

[45] Xu N , Tan GC, Wang HY. et al. Effect of biochar additions to soil on nitrogen leaching, microbial biomass and bacterial community structure. Eur J Soil Biol. 2016;74:1–8

[46] Adams RI , Miletto M, Taylor JW. et al. Dispersal in microbes: fungi in indoor air are dominated by outdoor air and show dispersal limitation at short distances. ISME J. 2013;7:1262–73 23426013 10.1038/ismej.2013.28 PMC3695294

[47] Caporaso JG , Kuczynski J, Stombaugh J. et al. QIIME allows analysis of high-throughput community sequencing data. Nat Methods. 2010;7:335–6 20383131 10.1038/nmeth.f.303 PMC3156573

[48] Schloss PD , Westcott SL, Ryabin T. et al. Introducing mothur: open-source, platform-independent, community-supported software for describing and comparing microbial communities. Appl Environ Microbiol. 2009;75:7537–41 19801464 10.1128/AEM.01541-09 PMC2786419

[49] Poly F , Monrozier LJ, Bally R. Improvement in the RFLP procedure for studying the diversity of *nifH* genes in communities of nitrogen fixers in soil. Res Microbiol. 2001;152:95–103 11281330 10.1016/s0923-2508(00)01172-4

[50] Zhou JZ , Wu LY, Deng Y. et al. Reproducibility and quantitation of amplicon sequencing-based detection. ISME J. 2011;5:1303–13 21346791 10.1038/ismej.2011.11 PMC3146266

