# Supplementary material for: Harnessing the power of microbes: Enhancing soybean growth in an acidic soil through AMF inoculation rather than P-fertilization
Source: Hortic Res. 2024 Mar 2;11(5):uhae067. doi: 10.1093/hr/uhae067 (PMC11079484; doi:10.1093/hr/uhae067)
Supplement: Web_Material_uhae067 [file web_material_uhae067.zip › Supplementary Information-R1.docx]

**Supplementary Information**

**Supplementary Figures**


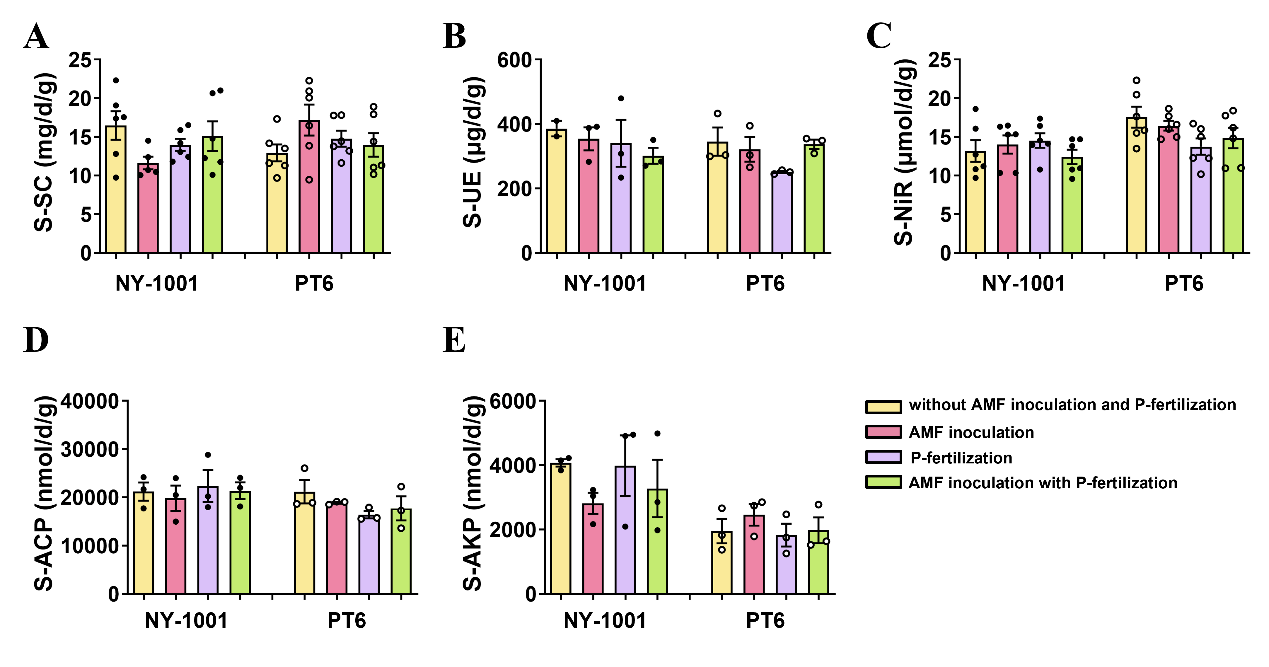


**Supplementary Figure 1. The activities of five key enzymes involved in the carbon, nitrogen and phosphorus cycle of root-associated microbial communities.** NY-1001 and PT6 in X-axis represent the lower P-efficiency recipient soybean ‘NY-1001’ and higher P-efficiency soybean ‘PT6’. S-SC, S-UE, S-NiR, S-ACP and S-AKP represent sucrase (A), urease (B), nitrite reductase (C), acid phosphatase (D) and alkaline phosphatase (E), respectively. The significance test was performed by using one-way ANOVA.


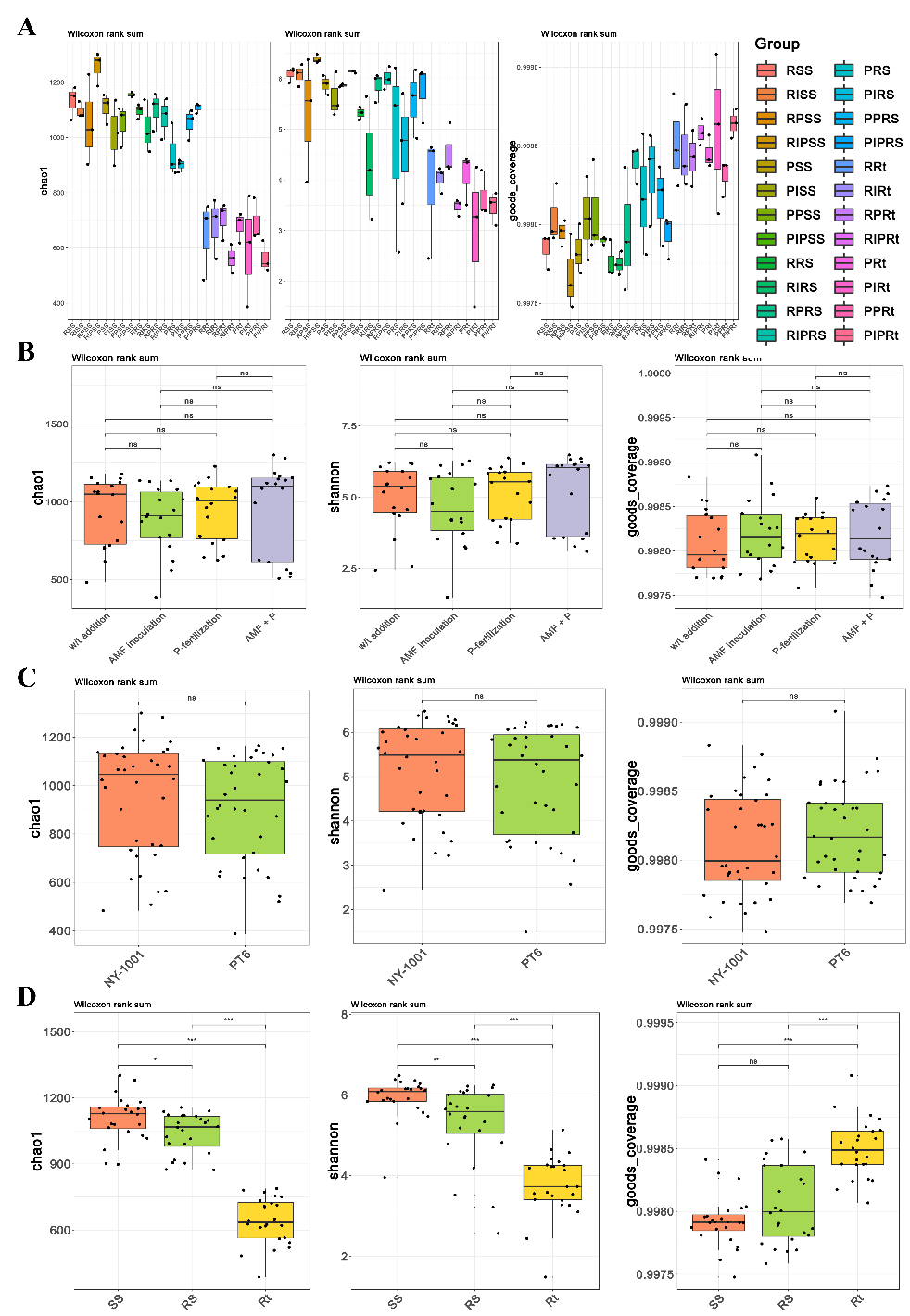


**Supplementary Figure 2. The boxplot of alpha diversity of root-associated fungal community.** (A)The Chao value, Shannon value and Good’s coverage of fungal community of all groups. The first letter R and P represent the low P-efficiency recipient soybean ‘NY-1001’ and high P-efficiency soybean PT6. I and P in the middle represent AMF inoculation and P-fertilization, respectively. SS, RS and Rt represent surrounding soils, rhizospheric soils, and intact roots, respectively. The boxplot of combined groups was mapped by using four different AMF inoculation and P-fertilization treatment groups (B), two different P-efficiency of soybean groups (C) and three different sampling compartment groups (D), respectively. Left, middle and right panels are Chao index, Shannon index and coverage index, respectively. Significance codes: * *p* < 0.05; ** *p* < 0.01; and *** *p* < 0.001.


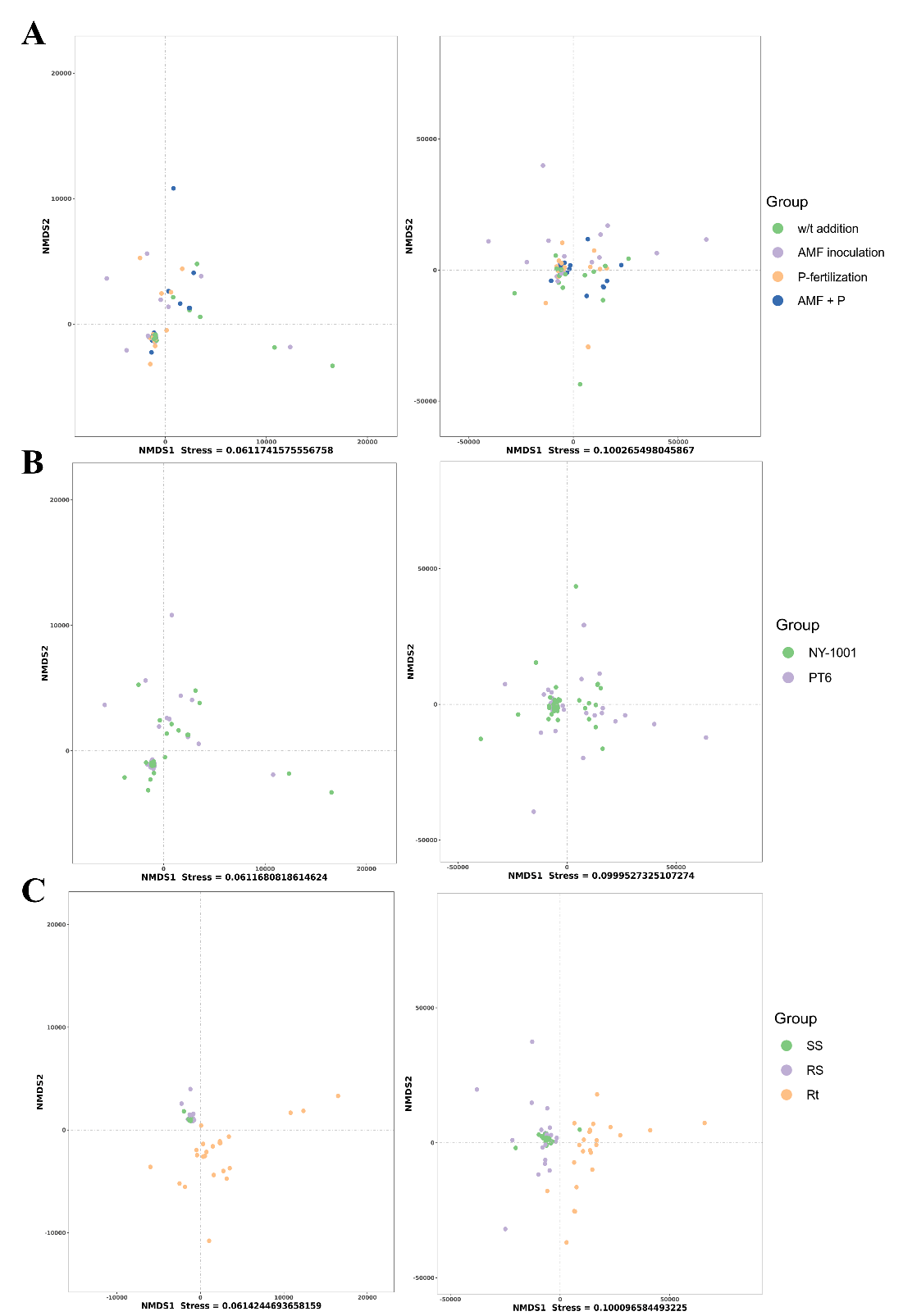


**Supplementary Figure 3.** **NMDS of microbial communities based on Euclidean distance.** (A) NMDS by mapping using four different treatment groups. (B) NMDS by mapping using two different P-efficiency of soybean groups. (C) NMDS by mapping using three different sampling compartment groups. Left panel is NMDS of bacterial community while right panel is NMDS of fungal community. ‘W/t addition’ and ‘AMF+P’ represent ‘no P-fertilization or AMF inoculation’ and ‘co-application of AMF and P-fertilizer’, respectively. NY-1001 and PT6 represent the low P-efficiency recipient soybean ‘NY-1001’ and high P-efficiency soybean PT6. SS, RS and Rt represent surrounding soils, rhizospheric soils, and intact roots, respectively.


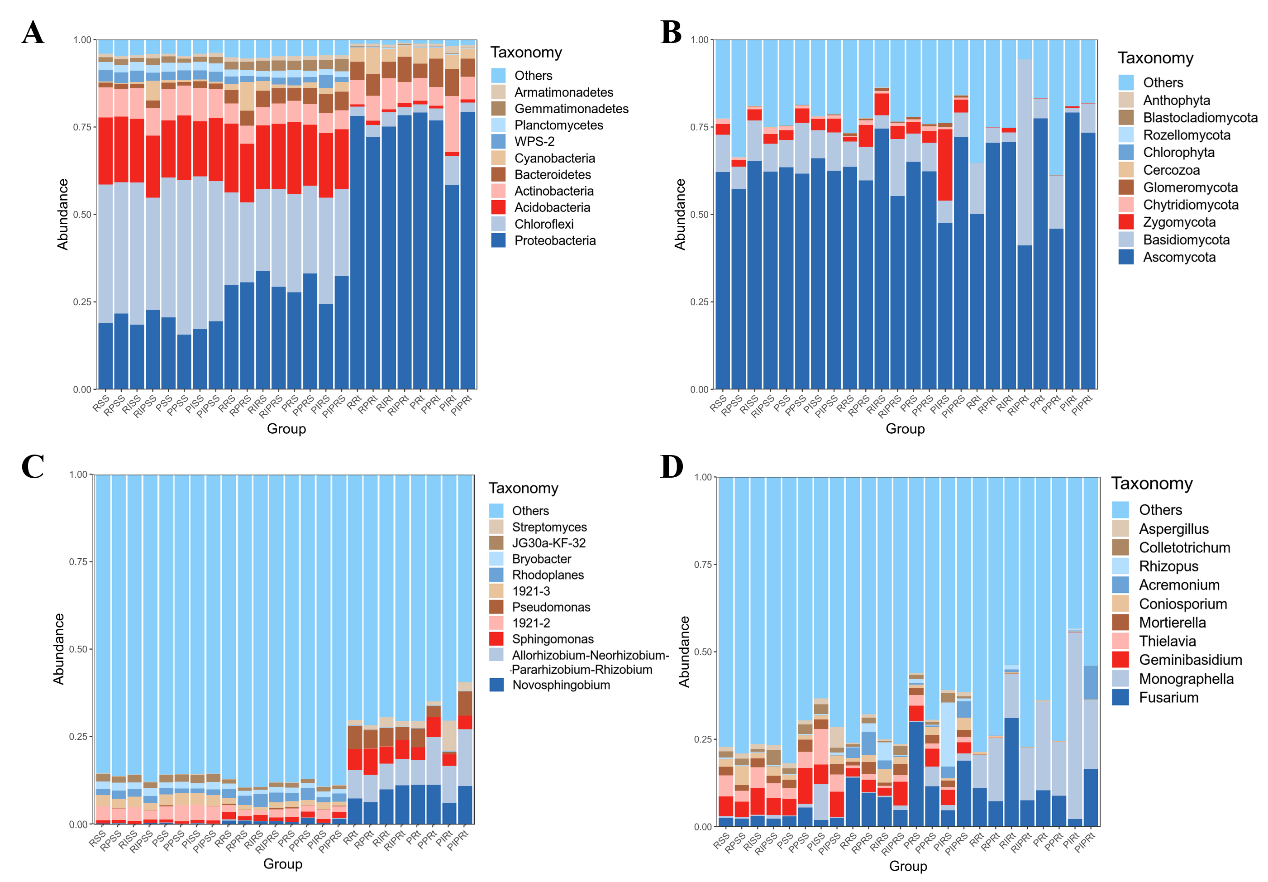


**Supplementary Figure 4. Relative abundances of microbial communities of samples at different taxonomic levels.** (A) The relative abundances of top 10 phyla of bacterial community. (B) The relative abundances of top 10 phyla of fungal community. (C) The relative abundances of top 10 genera of bacterial community. (D) The relative abundances of top 10 genera of fungal community. Treatments’ details were as in Supplementary Figure 2.


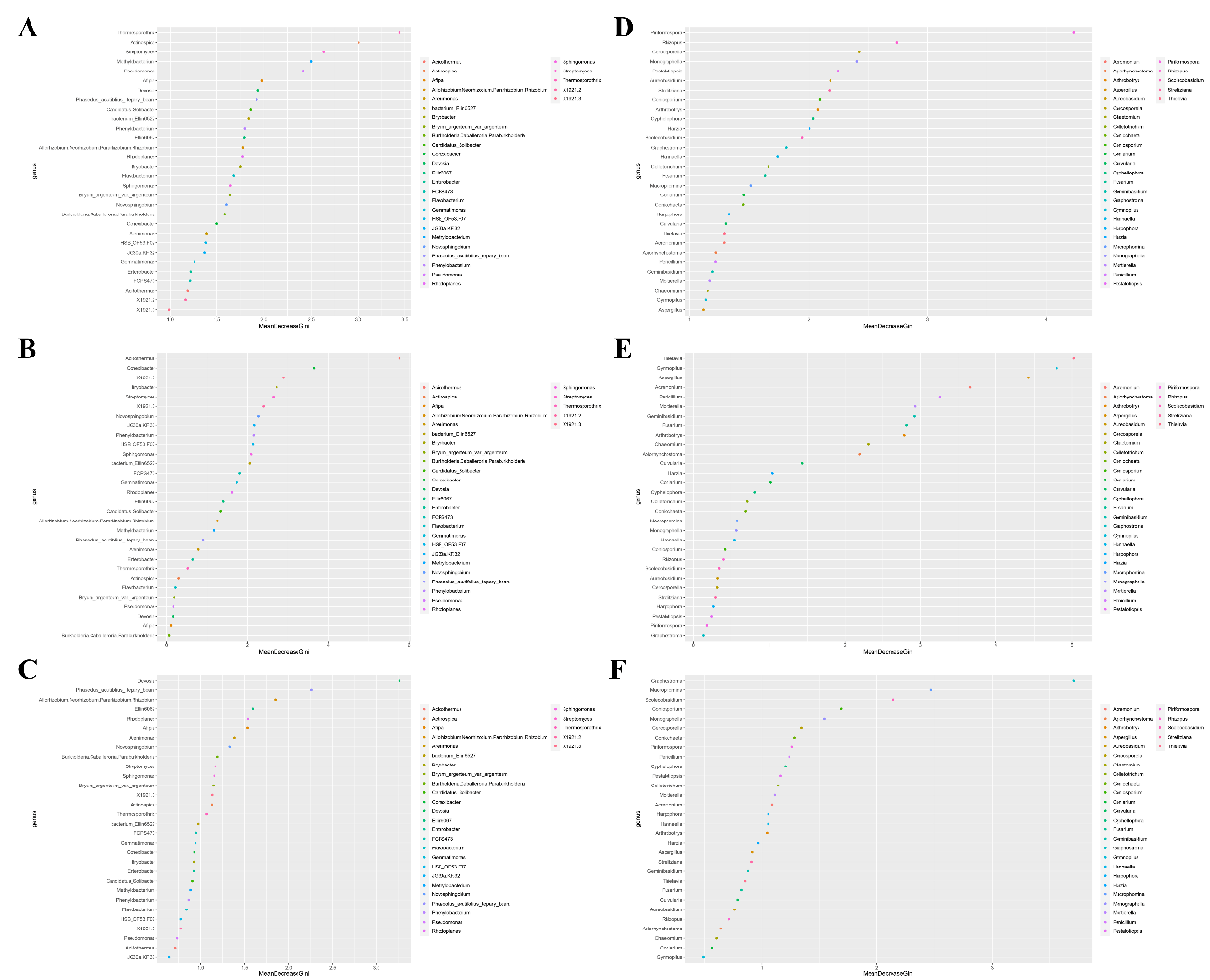


**Supplementary Figure 5. Thirty most important bacteria (A, B and C) and fungi (D, E and F) at the genus level correlated with different group based on the random forest model.** A and D correlated with the four different treatments, B and E correlated with the three different host niches, and C and F correlated with the two different soybeans based on the random forest model. A larger Mean Decrease Gini value corresponds to greater influence of the characteristic variable on the target.


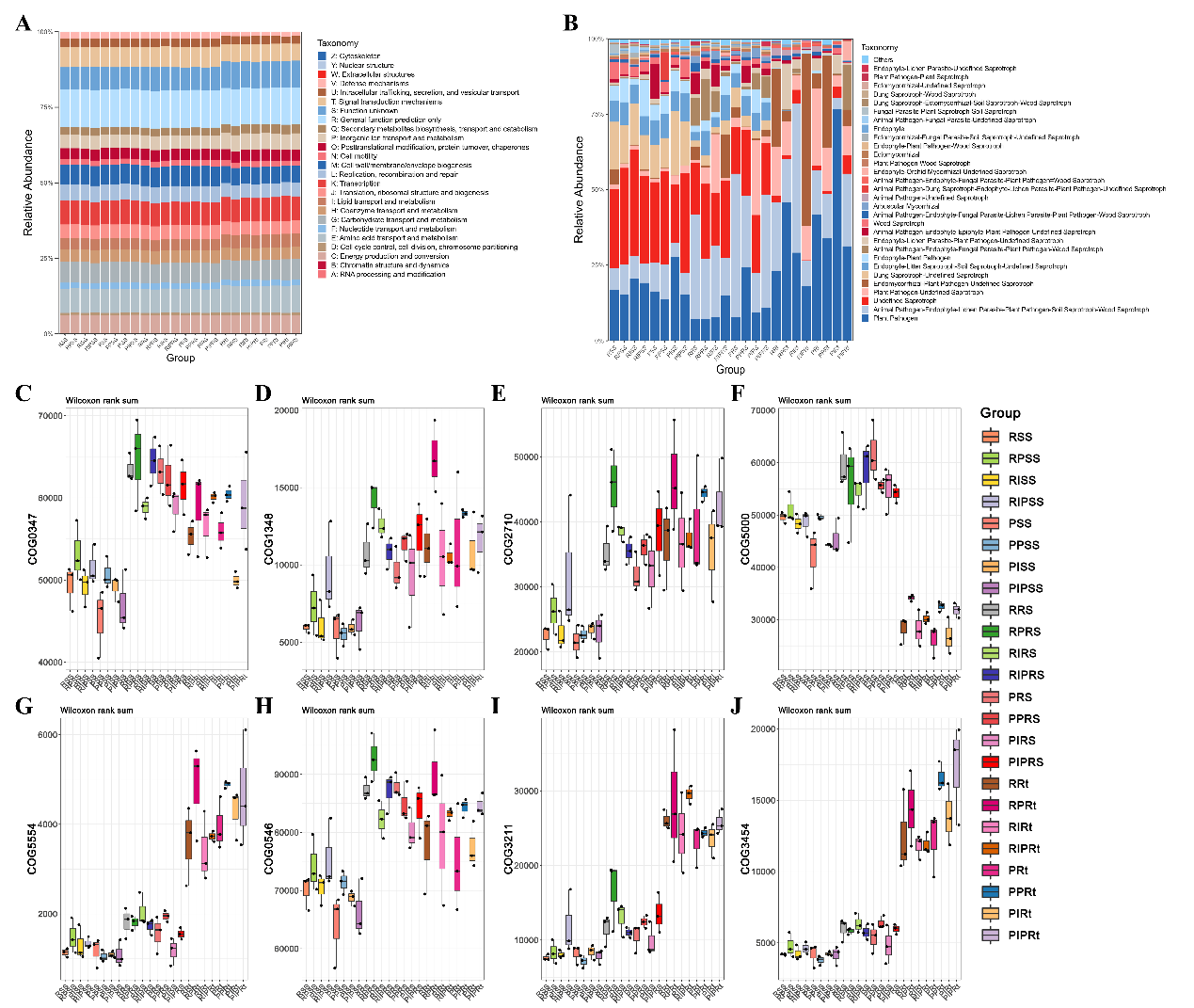


**Supplementary Figure 6. Functional prediction of microbial communities and the relative abundance of COG function classification which were directly related to nitrogen-fixation and phosphorus cycle.** Variations in composition of microbial functional groups inferred by PICRUSt2 (A) and FUNGuild (B). The five nitrogen-fixation COGs from (C) to (G) were described as nitrogen regulatory protein PII (C), nitrogenase subunit NifH (ATPase) (D), nitrogenase molybdenum-iron protein, alpha and beta chains (E), signal transduction histidine kinase involved in nitrogen fixation and metabolism regulation (F) and nitrogen fixation protein (G), respectively. The three COGs from (H) to (J) directly related to soil phosphorus cycle were described as predicted phosphatases (H), predicted phosphatase (I) and metal-dependent hydrolase involved in phosphonate metabolism (J), respectively. Significance codes: * *p* < 0.05; ** *p* < 0.01; and *** *p* < 0.001. Treatments’ details were as in Supplementary Figure 2.


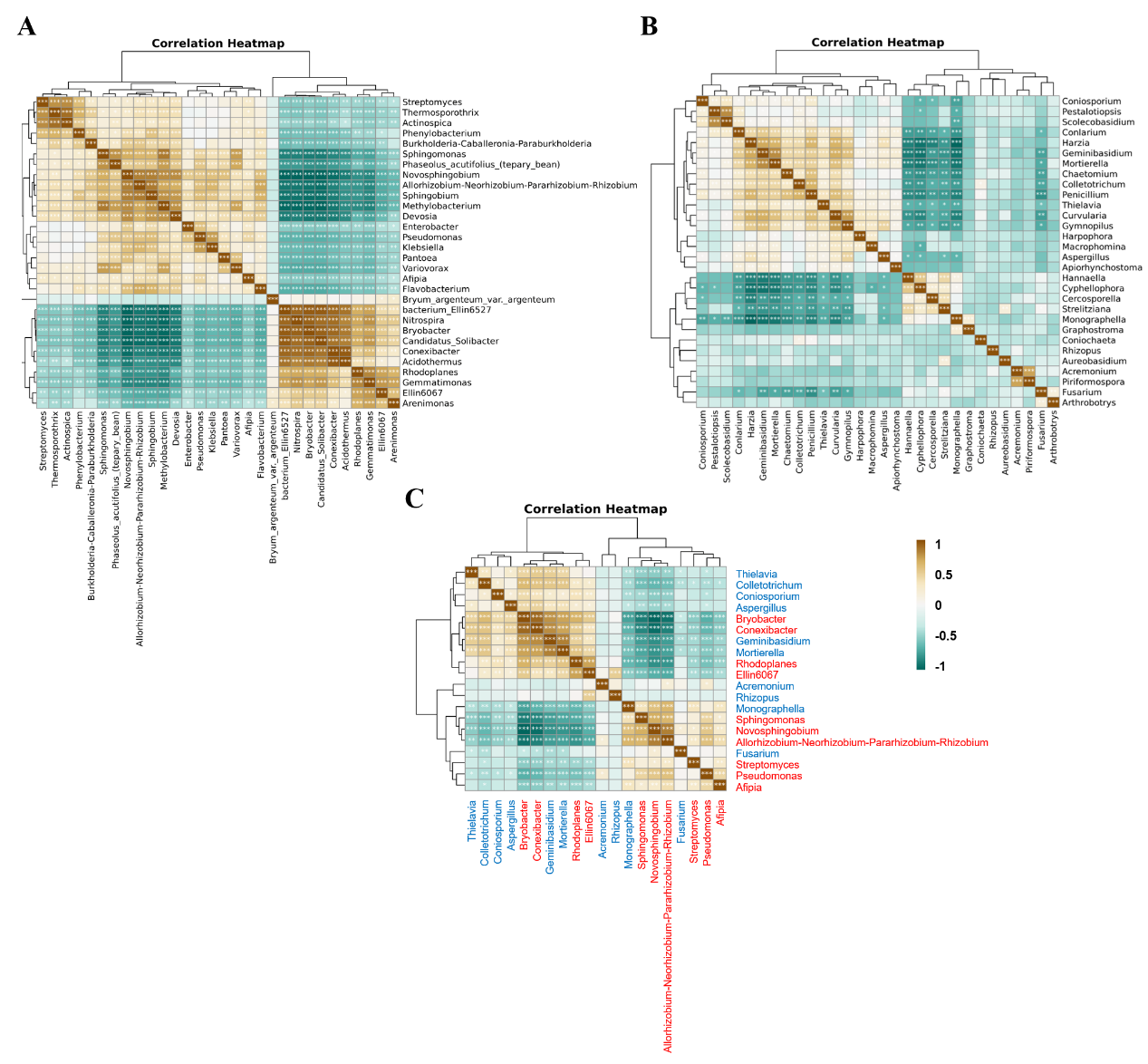


**Supplementary Figure 7. Pearson correlation analysis of the major 30 bacterial genera (A)，major 30 fungal genera (B) and 10 major bacterial and fungal genera (C) associated with the rhizosphere soils of soybean.** Brown represents a positive correlation (*r*>0), while green represents a negative correlation (*r*<0). **P* < 0.05, ***P* < 0.01, and ****P* < 0.001 indicate significant correlations. The red font and blue font in (C) represent bacterial genera and fungal genera, respectively.


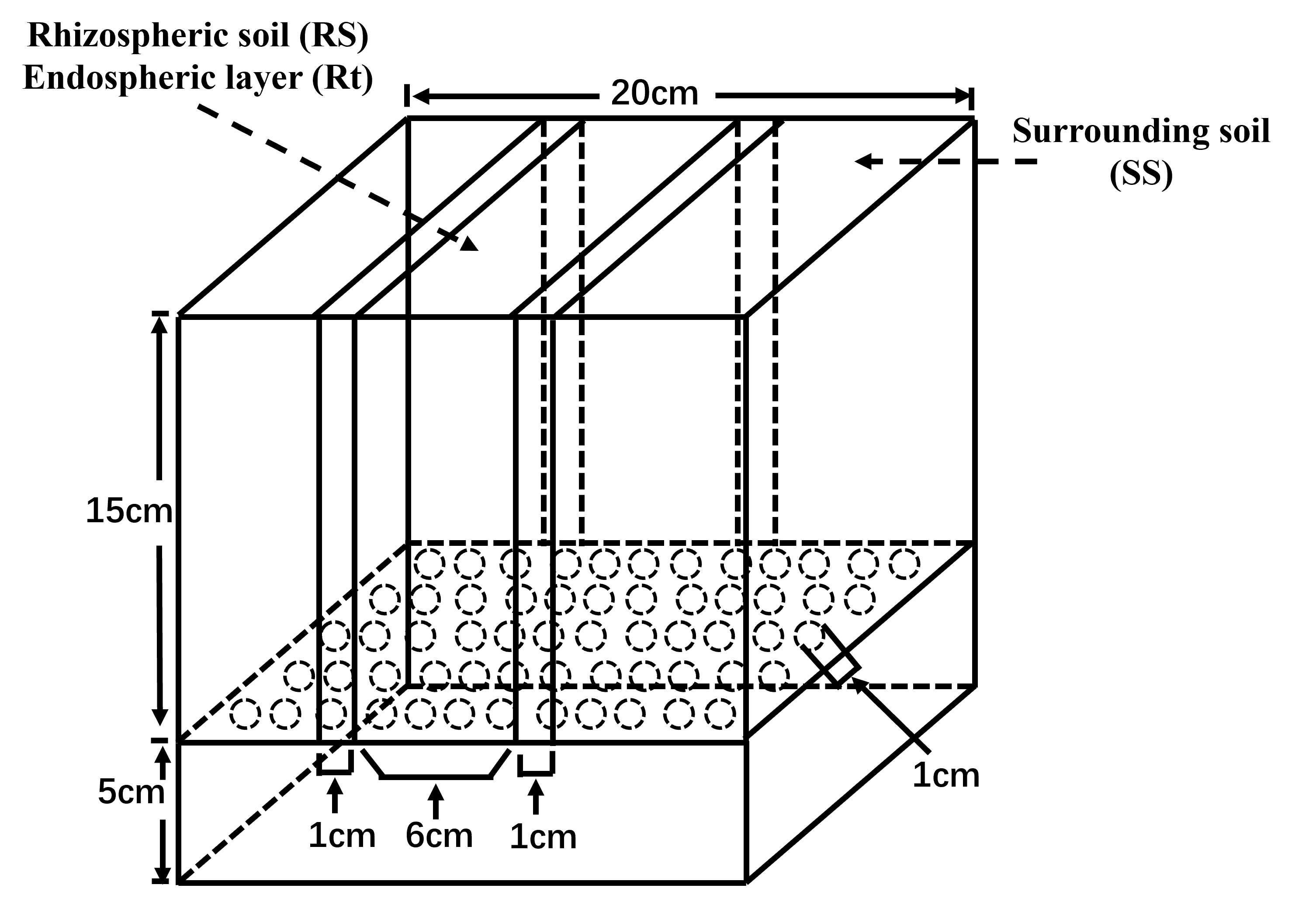


**Supplementary Figure 8. Schematic diagram of rhizobox.** The length*width*height of the rhizobox is 20cm*15cm*20cm. Two layers of nylon mesh are used to separate every two compartments to prevent roots from growing into the compartments on both sides. Soybeans were planted in the central compartment of the rhizobox.

**Supplementary Tables**

**Supplementary Table 1. Statistical analyses of root-associated bacterial community.**

| **Group vs. Group** | **Adonis** | | **ANOSIM** | |
| --- | --- | --- | --- | --- |
|  | **R^2^** | ***P-*value** | **Statistic** | ***P*-value** |
| RSS vs RPSS | 0.2204 | 0.3 | 0.2963 | 0.091 |
| RSS vs RISS | 0.20565 | 0.5 | 0.11111 | 0.189 |
| RSS vs RIPSS | 0.28646 | 0.1 | 0.33333 | 0.108 |
| RPSS vs RISS | 0.22558 | 0.2 | 0.11111 | 0.309 |
| RPSS vs RIPSS | 0.22975 | 0.3 | 0.11111 | 0.412 |
| RISS vs RIPSS | 0.27484 | 0.1 | 0.33333 | 0.087 |
| PSS vs PPSS | 0.2475 | 0.4 | 0.22222 | 0.306 |
| PSS vs PISS | 0.20215 | 0.6 | 0.11111 | 0.302 |
| PSS vs PIPSS | 0.18027 | 0.6 | -0.11111 | 0.813 |
| PPSS vs PISS | 0.29434 | 0.1 | 0.74074 | 0.123 |
| PPSS vs PIPSS | 0.27924 | 0.1 | 0.37037 | 0.113 |
| PISS vs PIPSS | 0.21104 | 0.3 | 0.07407 | 0.375 |
| RRS vs RPRS | 0.17823 | 0.8 | 0.03704 | 0.514 |
| RRS vs RIRS | 0.29236 | 0.1 | 0.48148 | 0.09 |
| RRS vs RIPRS | 0.23078 | 0.1 | 0.33333 | 0.098 |
| RPRS vs RIRS | 0.21667 | 0.5 | 0.07407 | 0.509 |
| RPRS vs RIPRS | 0.18796 | 0.7 | -0.11111 | 0.897 |
| RIRS vs RIPRS | 0.27987 | 0.1 | 0.55556 | 0.105 |
| PRS vs PPRS | 0.23614 | 0.2 | 0.07407 | 0.385 |
| PRS vs PIRS | 0.16875 | 0.6 | -0.11111 | 0.693 |
| PRS vs PIPRS | 0.30674 | 0.2 | 0.37037 | 0.193 |
| PPRS vs PIRS | 0.21895 | 0.3 | 0.18519 | 0.294 |
| PPRS vs PIPRS | 0.20768 | 0.4 | 0.18519 | 0.268 |
| PIRS vs PIPRS | 0.21514 | 0.3 | 0.11111 | 0.295 |
| RRt vs RPRt | 0.15227 | 0.8 | -0.37037 | 1 |
| RRt vs RIRt | 0.13192 | 0.9 | -0.25926 | 0.815 |
| RRt vs RIPRt | 0.42594 | 0.1 | 0.40741 | 0.095 |
| RPRt vs RIRt | 0.19983 | 0.5 | -0.03704 | 0.501 |
| RPRt vs RIPRt | 0.39988 | 0.1 | 0.22222 | 0.085 |
| RIRt vs RIPRt | 0.40298 | 0.1 | 0.22222 | 0.09 |
| PRt vs PPRt | 0.34284 | 0.1 | 0.40741 | 0.21 |
| PRt vs PIRt | 0.34348 | 0.1 | 0.59259 | 0.105 |
| PRt vs PIPRt | 0.27626 | 0.1 | 0.44444 | 0.097 |
| PPRt vs PIRt | 0.33975 | 0.1 | 0.44444 | 0.106 |
| PPRt vs PIPRt | 0.28843 | 0.1 | 0.11111 | 0.296 |
| PIRt vs PIPRt | 0.31049 | 0.1 | 0.44444 | 0.078 |
| RSS vs PSS | 0.20825 | 0.5 | 0.18519 | 0.197 |
| RPSS vs PPSS | 0.30686 | 0.1 | 0.51852 | 0.109 |
| RISS vs PISS | 0.24072 | 0.1 | 0.51852 | 0.094 |
| RIPSS vs PIPSS | 0.23129 | 0.2 | 0.14815 | 0.374 |
| RRS vs PRS | 0.19089 | 0.5 | 0.03704 | 0.413 |
| RPRS vs PPRS | 0.16964 | 1 | -0.07407 | 0.816 |
| RIRS vs PIRS | 0.2533 | 0.3 | 0.33333 | 0.311 |
| RIPRS vs PIPRS | 0.17899 | 0.8 | 0.03704 | 0.398 |
| RRt vs PRt | 0.23015 | 0.4 | 0.14815 | 0.307 |
| RPRt vs PPRt | 0.23173 | 0.2 | -0.07407 | 0.783 |
| RIRt vs PIRt | 0.31335 | 0.1 | 0.55555 | 0.088 |
| RIPRt vs PIPRt | 0.42227 | 0.1 | 0.22222 | 0.091 |
| RSS vs RRS | 0.44036 | 0.1 | 0.77778 | 0.112 |
| RSS vs RRt | 0.76248 | 0.1 | 1 | 0.096 |
| RRS vs RRt | 0.70502 | 0.1 | 1 | 0.103 |
| RPSS vs RPRS | 0.33473 | 0.1 | 0.55556 | 0.111 |
| RPSS vs RPRt | 0.70078 | 0.1 | 1 | 0.107 |
| RPRS vs RPRt | 0.59799 | 0.1 | 1 | 0.095 |
| RISS vs RIRS | 0.50856 | 0.1 | 0.96296 | 0.109 |
| RISS vs RIRt | 0.76172 | 0.1 | 1 | 0.101 |
| RIRS vs RIRt | 0.68409 | 0.1 | 1 | 0.109 |
| RIPSS vs RIPRS | 0.27441 | 0.1 | 0.37037 | 0.096 |
| RIPSS vs RIPRt | 0.88339 | 0.1 | 1 | 0.104 |
| RIPRS vs RIPRt | 0.88075 | 0.1 | 1 | 0.107 |
| PSS vs PRS | 0.29092 | 0.2 | 0.48148 | 0.203 |
| PSS vs PRt | 0.75416 | 0.1 | 1 | 0.099 |
| PRS vs PRt | 0.76164 | 0.1 | 1 | 0.094 |
| PPSS vs PPRS | 0.558 | 0.1 | 1 | 0.108 |
| PPSS vs PPRt | 0.87542 | 0.1 | 1 | 0.091 |
| PPRS vs PPRt | 0.81968 | 0.1 | 1 | 0.097 |
| PISS vs PIRS | 0.32032 | 0.1 | 0.25926 | 0.208 |
| PISS vs PIRt | 0.76768 | 0.1 | 1 | 0.116 |
| PIRS vs PIRt | 0.67118 | 0.1 | 1 | 0.103 |
| PIPSS vs PIPRS | 0.46426 | 0.1 | 0.96296 | 0.111 |
| PIPSS vs PIPRt | 0.73574 | 0.1 | 1 | 0.094 |
| PIPRS vs PIPRt | 0.68041 | 0.1 | 1 | 0.095 |

ANOSIM means analysis of similarities and Adonis is used for PERMANOVA (permutational multivariate analysis of variance) based on the Bray–Curtis distance metrics. The p-values in bold indicate the significant difference (*p* < 0.05 (*)) between groups by the tests. The first letter R and P represent the low P-efficiency recipient soybean cultivar ‘NY-1001’ and high P-efficiency GM soybean PT6. P and I represent phosphorus fertilization and AMF inoculation, respectively. SS, RS and Rt represent surrounding soils, rhizospheric soils, and intact roots, respectively.**Supplementary Table 2. Statistical analyses of root-associated fungal community.**

| **Group vs. Group** | **Adonis** | | **ANOSIM** | |
| --- | --- | --- | --- | --- |
|  | **R^2^** | ***P-*value** | **Statistic** | ***P*-value** |
| RSS vs RPSS | 0.25081 | 0.1 | 0.14814 | 0.277 |
| RSS vs RISS | 0.23381 | 0.2 | 0.14815 | 0.289 |
| RSS vs RIPSS | 0.26241 | 0.1 | 0.14815 | 0.194 |
| RPSS vs RISS | 0.27288 | 0.1 | 0.37037 | 0.09 |
| RPSS vs RIPSS | 0.26326 | 0.1 | 0.03704 | 0.525 |
| RISS vs RIPSS | 0.29084 | 0.1 | 0.07407 | 0.488 |
| PSS vs PPSS | 0.22164 | 0.2 | 0.11111 | 0.192 |
| PSS vs PISS | 0.24629 | 0.1 | 0.18518 | 0.087 |
| PSS vs PIPSS | 0.25084 | 0.1 | 0.14814 | 0.19 |
| PPSS vs PISS | 0.23899 | 0.2 | 0.2963 | 0.224 |
| PPSS vs PIPSS | 0.21272 | 0.4 | -0.14815 | 0.912 |
| PISS vs PIPSS | 0.29758 | 0.1 | 0.33333 | 0.103 |
| RRS vs RPRS | 0.23647 | 0.4 | 0.1481 | 0.272 |
| RRS vs RIRS | 0.32645 | 0.1 | 0.44444 | 0.093 |
| RRS vs RIPRS | 0.3839 | 0.1 | 0.59259 | 0.114 |
| RPRS vs RIRS | 0.3343 | 0.1 | 0.55556 | 0.199 |
| RPRS vs RIPRS | 0.27039 | 0.2 | 0.22222 | 0.209 |
| RIRS vs RIPRS | 0.44717 | 0.1 | 0.77778 | 0.102 |
| PRS vs PPRS | 0.20972 | 0.2 | 0 | 0.422 |
| PRS vs PIRS | 0.21942 | 0.3 | 0.1111 | 0.306 |
| PRS vs PIPRS | 0.24038 | 0.2 | 0.11111 | 0.192 |
| PPRS vs PIRS | 0.16754 | 0.7 | -0.18519 | 1 |
| PPRS vs PIPRS | 0.21191 | 0.6 | -0.03704 | 0.596 |
| PIRS vs PIPRS | 0.21399 | 0.5 | 0.03704 | 0.494 |
| RRT vs RPRT | 0.21916 | 0.2 | 0.07407 | 0.302 |
| RRT vs RIRT | 0.23043 | 0.3 | 0.14815 | 0.291 |
| RRT vs RIPRT | 0.53446 | 0.1 | 0.40741 | 0.104 |
| RPRT vs RIRT | 0.31029 | 0.1 | 0.2963 | 0.092 |
| RPRT vs RIPRT | 0.77878 | 0.1 | 1 | 0.1 |
| RIRT vs RIPRT | 0.67795 | 0.1 | 1 | 0.098 |
| PRT vs PPRT | 0.34393 | 0.2 | 0.48148 | 0.195 |
| PRT vs PIRT | 0.30037 | 0.2 | 0.33333 | 0.211 |
| PRT vs PIPRT | 0.15246 | 0.9 | -0.14815 | 0.828 |
| PPRT vs PIRT | 0.4504 | 0.1 | 0.51852 | 0.189 |
| PPRT vs PIPRT | 0.25653 | 0.3 | 0 | 0.395 |
| PIRT vs PIPRT | 0.27164 | 0.3 | 0.14815 | 0.278 |
| RSS vs PSS | 0.19854 | 0.5 | 0.03703 | 0.591 |
| RPSS vs PPSS | 0.26081 | 0.1 | 0.2963 | 0.114 |
| RISS vs PISS | 0.28919 | 0.1 | 0.59259 | 0.112 |
| RIPSS vs PIPSS | 0.32104 | 0.1 | 0.25926 | 0.191 |
| RRS vs PRS | 0.24148 | 0.3 | 0.14815 | 0.294 |
| RPRS vs PPRS | 0.20343 | 0.4 | 0 | 0.489 |
| RIRS vs PIRS | 0.28331 | 0.2 | 0.25926 | 0.209 |
| RIPRS vs PIPRS | 0.33935 | 0.1 | 0.55556 | 0.12 |
| RRT vs PRT | 0.20496 | 0.5 | 0.03704 | 0.498 |
| RPRT vs PPRT | 0.47285 | 0.1 | 0.77778 | 0.108 |
| RIRT vs PIRT | 0.37299 | 0.1 | 0.40741 | 0.084 |
| RIPRT vs PIPRT | 0.49026 | 0.1 | 0.44444 | 0.108 |
| RSS vs RRS | 0.46998 | 0.1 | 0.77778 | 0.086 |
| RSS vs RRT | 0.58816 | 0.1 | 0.96296 | 0.098 |
| RRS vs RRT | 0.41126 | 0.1 | 0.48148 | 0.092 |
| RPSS vs RPRS | 0.27227 | 0.1 | 0.22222 | 0.096 |
| RPSS vs RPRT | 0.65305 | 0.1 | 1 | 0.09 |
| RPRS vs RPRT | 0.63298 | 0.1 | 1 | 0.111 |
| RISS vs RIRS | 0.51105 | 0.1 | 0.88889 | 0.101 |
| RISS vs RIRT | 0.66611 | 0.1 | 1 | 0.096 |
| RIRS vs RIRT | 0.51071 | 0.1 | 1 | 0.11 |
| RIPSS vs RIPRS | 0.29205 | 0.1 | 0.33333 | 0.089 |
| RIPSS vs RIPRT | 0.89042 | 0.1 | 1 | 0.122 |
| RIPRS vs RIPRT | 0.85739 | 0.1 | 1 | 0.085 |
| PSS vs PRS | 0.30506 | 0.2 | 0.22222 | 0.289 |
| PSS vs PRT | 0.69187 | 0.1 | 1 | 0.096 |
| PRS vs PRT | 0.61775 | 0.1 | 1 | 0.104 |
| PPSS vs PPRS | 0.29786 | 0.1 | 0.48148 | 0.103 |
| PPSS vs PPRT | 0.74155 | 0.1 | 1 | 0.095 |
| PPRS vs PPRT | 0.64245 | 0.1 | 1 | 0.092 |
| PISS vs PIRS | 0.26414 | 0.1 | 0.18519 | 0.2 |
| PISS vs PIRT | 0.59055 | 0.1 | 0.88889 | 0.097 |
| PIRS vs PIRT | 0.50364 | 0.1 | 1 | 0.083 |
| PIPSS vs PIPRS | 0.43264 | 0.1 | 0.62963 | 0.101 |
| PIPSS vs PIPRT | 0.6276 | 0.1 | 1 | 0.085 |
| PIPRS vs PIPRT | 0.41927 | 0.1 | 0.77777 | 0.095 |

Treatments’ details were as in Supplementary Table 1.

**Supplementary Table 3. The relative abundance of major phyla in each sample (%).**

|  | **relative abundance (%)** | **NY-1001.SS** | | | |  | **PT6.SS** | | | |
| --- | --- | --- | --- | --- | --- | --- | --- | --- | --- | --- |
|  |  | **CK** | **RI** | **P** | **P+RI** |  | **CK** | **RI** | **P** | **P+RI** |
| Bacterial community | Proteobacteria | 18.85±1.22 | 18.51±2.63 | 21.66±4.91 | 22.71±2.85 |  | 20.53±8.4 | 17.21±2.13 | 15.65±0.34 | 19.48±4.57 |
|  | Chloroflexi | 39.72±0.65 | 40.63±2.77 | 37.56±6.59 | 32.07±3.04 |  | 40.01±8.76 | 43.66±2.85 | 44.2±1.18 | 40.02±5.53 |
|  | Acidobacteria | 19.17±0.59 | 18.21±0.66 | 18.79±0.58 | 17.75±2.83 |  | 16.32±1.11 | 15.77±0.59 | 18.46±1.01 | 18.04±0.75 |
|  | Actinobacteria | 8.57±0.22 | 8.69±0.77 | 7.78±0.58 | 7.87±1.3 |  | 9.02±1.08 | 9.58±0.7 | 8.5±1 | 8.37±0.38 |
|  | Bacteroidetes | 1.35±0.04 | 1.3±0.26 | 1.59±0.72 | 2.14±0.51 |  | 1.78±1.27 | 1.83±0.77 | 1.04±0.12 | 1.54±0.42 |
|  | Firmicutes | 0.28±0.01 | 0.3±0.09 | **0.31±0.03** | 0.27±0.05 |  | 0.27±0.04 | 0.32±0.03 | **0.27±0.02** | 0.27±0.01 |
| Fungal community | Ascomycota | 62.09±2.93 | 65.21±3.35 | 57.21±11.66 | 62.26±3.23 |  | 63.49±5.76 | 66.05±6.53 | 61.65±6.37 | 62.48±5.57 |
|  | Basidiomycota | 10.7±1.31 | 11.65±1.15 | 6.45±3.29 | 7.98±1.22 |  | 7.82±0.29 | 8.06±0.5 | 14.55±4.7 | 10.96±2.31 |
|  | Zygomycota | 3.07±0.91 | 3.17±0.28 | 1.99±1.15 | **2.78±0.62** |  | 2.81±0.6 ^B^ | 3.2±0.16 ^AB^ | 4.13±0.99 ^AB^ | **3.98±0.66 ^A^** |
|  | Chytridiomycota | 1.42±0.55 | 0.77±0.07 | 0.6±0.39 | 1.79±0.86 |  | 1.08±0.63 | 0.63±0.33 | 0.84±0.4 | 0.96±0.11 |
|  | Glomeromycota | 0.02±0.03 | 0.11±0.1 | 0.01±0.01 | 0.01±0.01 |  | 0.08±0.06 | 0.05±0.03 | 0.03±0.01 | 0.18±0.21 |
|  | Cercozoa | 0.07±0.02 | 0.1±0.05 | 0.08±0.05 | 0.09±0.01 |  | 0.06±0.02 ^B^ | 0.08±0.06 ^AB^ | 0.1±0.05 ^AB^ | 0.09±0.02 ^A^ |
|  | **relative abundance (%)** | **NY-1001.RS** | | | |  | **PT6.RS** | | | |
|  |  | **CK** | **RI** | **P** | **P+RI** |  | **CK** | **RI** | **P** | **P+RI** |
| Bacterial community | Proteobacteria | 0.2980±0.064 | 0.3388±0.042 | 0.3060±0.048 | 0.2931±0.031 |  | 0.2776±0.07 | 0.2433±0.078 | 0.3310±0.031 | 0.3244±0.021 |
|  | Chloroflexi | 0.2647±0.048 | 0.2346±0.06 | 0.2284±0.044 | 0.2797±0.027 |  | 0.2812±0.042 | 0.3049±0.058 | 0.2515±0.022 | 0.2487±0.024 |
|  | Acidobacteria | 0.1971±0.025 | 0.1811±0.018 | 0.168±0.044 | 0.1866±0.025 |  | 0.2048±0.036 | 0.1842±0.017 | 0.1738±0.002 | 0.1707±0.008 |
|  | Actinobacteria | 0.0575±0.006 | 0.0527±0.008 | 0.0515±0.001 | 0.0581±0.009 |  | 0.0616±0.007 | 0.0572±0.011 | 0.0587±0.003 | 0.0543±0.004 |
|  | Bacteroidetes | 0.0384±0.008 | 0.0463±0.013 | 0.0428±0.012 | 0.0433±0.01 |  | 0.0355±0.01 | 0.0545±0.028 | 0.0462±0.002 | 0.0531±0.004 |
|  | Firmicutes | 0.0027±0 | 0.0036±0.002 | **0.0032±0.001** | 0.0026±0 |  | 0.0027±0 | 0.0031±0 | **0.0024±0.001** | 0.0032±0.001 |
| Fungal community | Ascomycota | 63.59±2.65 ^ab^ | 74.52±13.64 ^a^ | 59.74±17.03 ^ab^ | 55.22±11.36 ^b^ |  | 65.06±13.48 | 47.51±15.38 | 62.31±8.7 | 72.21±3.26 |
|  | Basidiomycota | 7.23±1.93 | 3.86±1.51 | 9.61±7.81 | 16.33±8.81 |  | 8.08±5.65 | 6.46±3.06 | 8.12±3.39 | 6.91±1.57 |
|  | Zygomycota | 1.34±0.51 | 6.23±3.7 | 6.24±3.8 | 3.75±1.72 |  | 3.3±2.08 | 20.38±29.67 | 3.37±0.73 | 3.7±1.17 |
|  | Chytridiomycota | 0.27±0.11 | 0.73±0.82 | 1.38±1.56 | 0.83±0.22 |  | 0.74±0.49 | 0.7±0.57 | 1.36±1 | 0.61±0.32 |
|  | Glomeromycota | 0.77±0.7 | 0.89±1.23 | 0.42±0.34 | 0.3±0.17 |  | 0.59±0.53 | 1.04±1.03 | 0.64±0.6 | 0.57±0.17 |
|  | Cercozoa | 0.08±0.05 | 0.03±0.01 | 0.11±0.08 | 0.15±0.07 |  | 0.08±0.07 | 0.05±0.06 | 0.06±0.03 | 0.03±0.02 |
|  | **relative abundance (%)** | **NY-1001.Rt** | | | |  | **PT6.Rt** | | | |
|  |  | **CK** | **RI** | **P** | **P+RI** |  | **CK** | **RI** | **P** | **P+RI** |
| Bacterial community | Proteobacteria | 78.17±5.92 | 75.14±11.13 | 72.21±10.16 | 78.4±0.51 |  | 79.15±9.31 | 58.44±12.3 | 76.9±0.43 | 79.33±10.28 |
|  | Chloroflexi | 2.68±1.94 | 4.04±1.36 | 3.34±1.04 | 2.29±0.75 |  | 2.34±1.52 | 8.23±4.6 | 3.39±0.65 | 2.67±1.1 |
|  | Acidobacteria | 0.59±0.21 | 0.89±0.6 | 1.26±0.84 | 1.09±0.39 |  | 1.04±0.32 | 1.15±0.5 | 0.79±0.1 | 0.93±0.76 |
|  | Actinobacteria | 6.99±1.48 | 8.92±4.42 | 7.12±3.8 | 6.08±0.82 |  | 6.5±3.21 | 16.04±12.84 | 5.36±0.53 | 6.47±5.07 |
|  | Bacteroidetes | 5.24±1.47 | 4.68±0.92 | 6.18±1.53 | 7.19±0.64 |  | 4.12±1.23 | 7.69±5.52 | 8.1±2.74 | 5.15±1.24 |
|  | Firmicutes | 0.11±0.03 | 0.13±0.08 | 0.08±0.06 | 0.05±0.01 |  | 0.11±0.05 | 0.08±0.05 | 0.07±0.02 | 0.11±0 |
| Fungal community | Ascomycota | 50.13±31.99 ^ab^ | 70.69±12.31 ^ab^ | 70.47±5.15 ^a^ | 41.16±0.39 ^b^ |  | 77.49±12.48 | 79.17±19.49 | 45.94±21.16 | 73.38±15.5 |
|  | Basidiomycota | 14.35±16.03 ^ab^ | 2.77±1.55 ^b^ | 4.43±2.42 ^b^ | **53.18±2.09 ^a^** |  | 5.67±3.53 | 1.19±0.8 | 15.18±8.44 | **8.37±6.7** |
|  | Zygomycota | 0.04±0.03 | 1.27±0.99 | 0.12±0.11 | 0.01±0 |  | 0.03±0.01 | 0.53±0.84 | 0.03±0.02 | 0.04±0.01 |
|  | Chytridiomycota | 0.025±0.027 | 0.009±0.009 | 0.015±0.009 | 0.004±0.001 |  | 0.006±0.003 | 0.012±0.011 | 0.009±0.006 | 0.011±0.009 |
|  | Glomeromycota | 0.009±0.006 | 0.029±0.03 | 0.003±0.002 | 0.002±0.002 |  | 0.011±0.013 | 0.026±0.028 | 0.012±0.004 | 0.025±0.016 |
|  | Cercozoa | 0.001±0.001 | 0.008±0.001 | 0.006±0.005 | 0.005±0.003 |  | 0.001±0.001 | 0.002±0.003 | 0.003±0.004 | 0.003±0.003 |

NY-1001 and PT6 represent the low P-efficiency recipient soybean cultivar ‘NY-1001’ and high P-efficiency GM soybean PT6. SS, RS and Rt represent surrounding soils, rhizospheric soils, and intact roots, respectively. SD stands for standard deviation (n = 3). The significance test was performed by using one-way ANOVA. The values in bold indicate the significant difference (*p*< 0.05) between NY-1001 and PT6 groups, while the values in red without same superscript letter indicate the significant difference (*p*< 0.05) between treatments groups within NY-1001 or PT6 groups.

**Supplementary Table 4. The relative abundance of nitrogen-fixing bacterial genus in each sample (%).**

| **Taxonomy** | **RSS1** | **RSS2** | **RSS3** | **RPSS1** | **RPSS2** | **RPSS3** | **RISS1** | **RISS2** | **RISS3** | **RIPSS1** | **RIPSS2** | **RIPSS3** |
| --- | --- | --- | --- | --- | --- | --- | --- | --- | --- | --- | --- | --- |
| Mycobacterium | 0.005164 | 0.004427 | 0.005246 | 0.00367 | 0.007116 | 0.004601 | 0.004913 | 0.005565 | 0.004745 | 0.004179 | 0.004858 | 0.005791 |
| Burkholderia-Caballeronia-Paraburkholderia | 0.003464 | 0.001696 | 0.002729 | 0.002952 | 0.003629 | 0.004175 | 0.002536 | 0.001852 | 0.001609 | 0.005233 | 0.00315 | 0.002238 |
| Devosia | 0.002091 | 0.002704 | 0.002331 | 0.002589 | 0.003788 | 0.002588 | 0.002244 | 0.002345 | 0.002717 | 0.002342 | 0.001922 | 0.003629 |
| Streptomyces | 0.001464 | 0.001553 | 0.002056 | 0.001445 | 0.00139 | 0.001383 | 0.002085 | 0.002131 | 0.001242 | 0.001018 | 0.001628 | 0.0016 |
| Mesorhizobium | 0.000936 | 0.000625 | 0.001223 | 0.001011 | 0.001797 | 0.00156 | 0.001096 | 0.00094 | 0.000813 | 0.001261 | 0.001157 | 0.001981 |
| Bradyrhizobium | 0.000564 | 0.000428 | 0.000505 | 0.000293 | 0.001106 | 0.000798 | 0.000592 | 0.000624 | 0.0005 | 0.000486 | 0.000783 | 0.000686 |
| Bacillus | 0.000546 | 0.000384 | 0.000505 | 0.0007 | 0.000469 | 0.000461 | 0.000459 | 0.000344 | 0.000259 | 0.00036 | 0.000276 | 0.000457 |
| Allorhizobium-Neorhizobium-Pararhizobium-Rhizobium | 0.000336 | 0.000295 | 0.000346 | 0.000319 | 0.000797 | 0.000381 | 0.000468 | 0.00053 | 0.000429 | 0.000567 | 0.000383 | 0.000419 |
| Pseudomonas | 0.000309 | 0.000223 | 0.000868 | 0.000124 | 0.000186 | 0.000408 | 0.000292 | 0.000186 | 0.000402 | 0.000279 | 9.79E-05 | 0.00021 |
| Methylobacterium | 0.000191 | 0.000161 | 0.000151 | 6.21E-05 | 0.000115 | 0.000177 | 0.000212 | 9.31E-05 | 0.000652 | 0.000802 | 0.000445 | 0.000371 |
| Micromonospora | 0.000145 | 3.57E-05 | 7.98E-05 | 4.43E-05 | 0.000221 | 0.000257 | 2.65E-05 | 0.000121 | 0.000313 | 9.01E-05 | 0 | 5.71E-05 |
| Arthrobacter | 6.36E-05 | 8.93E-06 | 0.00016 | 0.000248 | 0.000159 | 0.000168 | 4.42E-05 | 7.45E-05 | 5.36E-05 | 0.000153 | 9.79E-05 | 6.67E-05 |
| Stenotrophomonas | 2.73E-05 | 0 | 3.54E-05 | 0 | 0 | 2.66E-05 | 1.77E-05 | 0 | 1.79E-05 | 9.01E-06 | 4.45E-05 | 1.90E-05 |
| Paeniclostridium | 9.09E-06 | 8.93E-06 | 1.77E-05 | 8.87E-06 | 8.85E-06 | 8.86E-06 | 8.84E-06 | 9.31E-06 | 0 | 1.80E-05 | 0 | 0 |
| Rhodopseudomonas | 0 | 8.93E-06 | 1.77E-05 | 8.87E-06 | 0 | 0 | 4.42E-05 | 9.31E-06 | 2.68E-05 | 6.30E-05 | 2.67E-05 | 0 |
| Ensifer | 0 | 0 | 0 | 8.87E-06 | 0 | 1.77E-05 | 0 | 0 | 8.94E-06 | 0 | 0 | 9.52E-06 |
| Agromyces | 0 | 0 | 0 | 0 | 0 | 8.86E-06 | 1.77E-05 | 0 | 8.94E-06 | 2.70E-05 | 0 | 9.52E-06 |
| Acinetobacter | 0 | 0 | 8.86E-06 | 8.87E-06 | 0 | 1.77E-05 | 0 | 1.86E-05 | 2.68E-05 | 9.01E-06 | 1.78E-05 | 0 |
| Azorhizobium | 0 | 0 | 0 | 0 | 0 | 0 | 0 | 0 | 0 | 0 | 0 | 9.52E-06 |
| **Taxonomy** | **PSS1** | **PSS2** | **PSS3** | **PPSS1** | **PPSS2** | **PPSS3** | **PISS1** | **PISS2** | **PISS3** | **PIPSS1** | **PIPSS2** | **PIPSS3** |
| Mycobacterium | 0.004108 | 0.005127 | 0.004145 | 0.004388 | 0.004662 | 0.004183 | 0.006153 | 0.005512 | 0.006105 | 0.004488 | 0.005803 | 0.003533 |
| Burkholderia-Caballeronia-Paraburkholderia | 0.001552 | 0.002383 | 0.002029 | 0.001963 | 0.002946 | 0.001898 | 0.00204 | 0.003281 | 0.002646 | 0.002148 | 0.001617 | 0.001903 |
| Devosia | 0.002608 | 0.002961 | 0.000769 | 0.001876 | 0.001069 | 0.001344 | 0.00102 | 0.002459 | 0.001009 | 0.001013 | 0.002274 | 0.001296 |
| Streptomyces | 0.001236 | 0.001553 | 0.001618 | 0.002085 | 0.001276 | 0.00145 | 0.002627 | 0.002476 | 0.001787 | 0.001737 | 0.00195 | 0.001631 |
| Mesorhizobium | 0.001067 | 0.001164 | 0.000393 | 0.00068 | 0.000647 | 0.00087 | 0.001003 | 0.000919 | 0.000549 | 0.000637 | 0.001246 | 0.000489 |
| Bradyrhizobium | 0.000359 | 0.000542 | 0.000446 | 0.000558 | 0.000512 | 0.000387 | 0.000527 | 0.00056 | 0.000389 | 0.000445 | 0.000733 | 0.000371 |
| Bacillus | 0.000253 | 0.000388 | 0.000236 | 0.000436 | 0.000467 | 0.000431 | 0.000467 | 0.000394 | 0.000416 | 0.000454 | 0.000561 | 0.000399 |
| Allorhizobium-Neorhizobium-Pararhizobium-Rhizobium | 0.00226 | 0.000749 | 0.000595 | 0.00041 | 0.000476 | 0.000343 | 0.000423 | 0.002301 | 0.00054 | 0.000585 | 0.000571 | 0.000498 |
| Pseudomonas | 0.007181 | 0.001444 | 0.000516 | 0.000393 | 0.000126 | 0.000308 | 0.000173 | 0.000114 | 0.000319 | 0.000454 | 0.000552 | 0.000254 |
| Methylobacterium | 0.001172 | 0.000406 | 0.000175 | 0.000201 | 0.000225 | 0.000132 | 0.000752 | 0.000726 | 0.000212 | 0.000454 | 0.000238 | 0.000226 |
| Micromonospora | 6.34E-05 | 7.22E-05 | 3.50E-05 | 5.23E-05 | 3.59E-05 | 6.15E-05 | 6.05E-05 | 4.37E-05 | 1.77E-05 | 3.49E-05 | 0.000219 | 5.44E-05 |
| Arthrobacter | 5.28E-05 | 0.000144 | 6.12E-05 | 5.23E-05 | 5.39E-05 | 3.51E-05 | 5.19E-05 | 0.000114 | 8.85E-06 | 8.73E-05 | 8.56E-05 | 5.44E-05 |
| Stenotrophomonas | 0.002059 | 0.000469 | 9.62E-05 | 8.72E-06 | 3.59E-05 | 8.79E-06 | 8.64E-06 | 8.75E-05 | 2.65E-05 | 1.75E-05 | 1.90E-05 | 0 |
| Paeniclostridium | 0 | 0 | 0 | 8.72E-06 | 1.80E-05 | 8.79E-06 | 0 | 8.75E-06 | 0 | 0 | 1.90E-05 | 9.06E-06 |
| Rhodopseudomonas | 2.11E-05 | 1.81E-05 | 2.62E-05 | 1.74E-05 | 0 | 0 | 8.64E-06 | 5.25E-05 | 1.77E-05 | 0 | 2.85E-05 | 3.62E-05 |
| Ensifer | 2.11E-05 | 0 | 8.74E-06 | 0 | 0 | 2.64E-05 | 8.64E-06 | 5.25E-05 | 8.85E-06 | 1.75E-05 | 1.90E-05 | 0 |
| Agromyces | 3.17E-05 | 1.81E-05 | 0 | 0 | 8.98E-06 | 0 | 0 | 0.000122 | 0 | 3.49E-05 | 0 | 0 |
| Acinetobacter | 2.11E-05 | 0.000126 | 8.74E-06 | 0 | 8.98E-06 | 0 | 1.73E-05 | 3.50E-05 | 0 | 0 | 0 | 0 |
| Azorhizobium | 2.11E-05 | 0 | 0 | 0 | 0 | 0 | 0 | 1.75E-05 | 0 | 0 | 0 | 0 |
| **Taxonomy** | **RRS1** | **RRS2** | **RRS3** | **RPRS1** | **RPRS2** | **RPRS3** | **RIRS1** | **RIRS2** | **RIRS3** | **RIPRS1** | **RIPRS2** | **RIPRS3** |
| Mycobacterium | 0.003521 | 0.003004 | 0.003329 | 0.00232 | 0.003744 | 0.003363 | 0.002871 | 0.004069 | 0.003873 | 0.003029 | 0.00241 | 0.003831 |
| Burkholderia-Caballeronia-Paraburkholderia | 0.002454 | 0.003255 | 0.004022 | 0.004192 | 0.016105 | 0.002089 | 0.003047 | 0.004213 | 0.005304 | 0.001147 | 0.002928 | 0.00165 |
| Devosia | 0.002738 | 0.005022 | 0.005623 | 0.002432 | 0.006607 | 0.004374 | 0.00331 | 0.007186 | 0.002743 | 0.003966 | 0.002608 | 0.00195 |
| Streptomyces | 0.000913 | 0.001184 | 0.000873 | 0.000655 | 0.000486 | 0.0009 | 0.001238 | 0.001114 | 0.000811 | 0.000639 | 0.001365 | 0.000949 |
| Mesorhizobium | 0.002273 | 0.002421 | 0.001925 | 0.001216 | 0.002478 | 0.001316 | 0.001879 | 0.001401 | 0.001467 | 0.000779 | 0.001356 | 0.000838 |
| Bradyrhizobium | 0.000878 | 0.001166 | 0.000639 | 0.000595 | 0.001009 | 0.000535 | 0.001001 | 0.001096 | 0.000993 | 0.000744 | 0.000846 | 0.00053 |
| Bacillus | 0.000138 | 0.000233 | 0.000288 | 0.000121 | 0.000239 | 0.000178 | 0.000307 | 0.000225 | 0.000319 | 0.000228 | 0.000147 | 0.000351 |
| Allorhizobium-Neorhizobium-Pararhizobium-Rhizobium | 0.000517 | 0.001211 | 0.010544 | 0.000431 | 0.002276 | 0.000603 | 0.000457 | 0.000799 | 0.001877 | 0.00063 | 0.002315 | 0.000368 |
| Pseudomonas | 0.000198 | 0.000717 | 0.007935 | 0.000319 | 0.001064 | 0.000374 | 0.000342 | 0.000656 | 0.000401 | 0.008781 | 0.001632 | 0.00118 |
| Methylobacterium | 0.000456 | 0.002448 | 0.000864 | 0.000155 | 0.001202 | 0.000306 | 0.000255 | 0.000467 | 0.002379 | 0.00014 | 0.000717 | 0.000239 |
| Micromonospora | 8.61E-06 | 0.00017 | 1.80E-05 | 2.59E-05 | 0.000174 | 8.49E-06 | 2.63E-05 | 0.000162 | 5.47E-05 | 0 | 8.64E-06 | 4.28E-05 |
| Arthrobacter | 2.58E-05 | 3.59E-05 | 0.000135 | 6.90E-05 | 0.000184 | 2.55E-05 | 0 | 6.29E-05 | 7.29E-05 | 0.00014 | 5.18E-05 | 7.70E-05 |
| Stenotrophomonas | 2.58E-05 | 8.97E-05 | 0.00493 | 6.90E-05 | 0.000229 | 5.10E-05 | 6.15E-05 | 7.19E-05 | 0.000283 | 1.75E-05 | 6.91E-05 | 1.71E-05 |
| Paeniclostridium | 0 | 0 | 0 | 0 | 0 | 8.49E-06 | 8.78E-06 | 0 | 9.11E-06 | 0 | 0 | 0 |
| Rhodopseudomonas | 3.44E-05 | 0.000135 | 0.000324 | 3.45E-05 | 0.000138 | 4.25E-05 | 1.76E-05 | 2.69E-05 | 7.29E-05 | 2.63E-05 | 5.18E-05 | 0 |
| Ensifer | 0 | 2.69E-05 | 1.80E-05 | 1.72E-05 | 4.59E-05 | 5.10E-05 | 0 | 0 | 0 | 0 | 0.000216 | 1.71E-05 |
| Agromyces | 7.75E-05 | 4.48E-05 | 1.80E-05 | 0 | 2.75E-05 | 6.79E-05 | 0 | 2.69E-05 | 0.000155 | 1.75E-05 | 0 | 1.71E-05 |
| Acinetobacter | 8.61E-06 | 4.48E-05 | 0 | 0 | 0 | 2.55E-05 | 0 | 8.98E-06 | 0 | 0 | 1.73E-05 | 0.000428 |
| Azorhizobium | 0 | 0 | 0.00018 | 0 | 0 | 0 | 8.78E-06 | 0 | 3.65E-05 | 0 | 0 | 0 |
| **Taxonomy** | **PRS1** | **PRS2** | **PRS3** | **PPRS1** | **PPRS2** | **PPRS3** | **PIRS1** | **PIRS2** | **PIRS3** | **PIPRS1** | **PIPRS2** | **PIPRS3** |
| Mycobacterium | 0.003541 | 0.004285 | 0.003235 | 0.003164 | 0.003034 | 0.003344 | 0.002427 | 0.003105 | 0.002637 | 0.003955 | 0.003286 | 0.003327 |
| Burkholderia-Caballeronia-Paraburkholderia | 0.002734 | 0.00453 | 0.00229 | 0.001306 | 0.003658 | 0.004467 | 0.004159 | 0.001198 | 0.004569 | 0.001253 | 0.00356 | 0.004817 |
| Devosia | 0.001497 | 0.004758 | 0.004389 | 0.005435 | 0.0027 | 0.003953 | 0.000686 | 0.000822 | 0.001497 | 0.002831 | 0.002568 | 0.0019 |
| Streptomyces | 0.001279 | 0.000841 | 0.000971 | 0.000771 | 0.002304 | 0.001323 | 0.00123 | 0.000822 | 0.000783 | 0.001458 | 0.002338 | 0.002203 |
| Mesorhizobium | 0.000782 | 0.00304 | 0.001353 | 0.001438 | 0.003166 | 0.00276 | 0.001523 | 0.000805 | 0.002167 | 0.001132 | 0.001479 | 0.001766 |
| Bradyrhizobium | 0.000597 | 0.000885 | 0.000867 | 0.000728 | 0.00175 | 0.001445 | 0.000569 | 0.000534 | 0.000809 | 0.000498 | 0.00077 | 0.000749 |
| Bacillus | 0.000143 | 0.000307 | 0.000208 | 0.00014 | 0.000132 | 0.000331 | 0.000159 | 0.000219 | 0.000148 | 0.000214 | 0.000381 | 0.000241 |
| Allorhizobium-Neorhizobium-Pararhizobium-Rhizobium | 0.00037 | 0.000946 | 0.000642 | 0.001061 | 0.001425 | 0.000792 | 0.000619 | 0.000499 | 0.00087 | 0.002677 | 0.003188 | 0.001873 |
| Pseudomonas | 0.000261 | 0.008026 | 0.000651 | 0.000964 | 0.000378 | 0.000313 | 0.000209 | 0.000175 | 0.000844 | 0.000772 | 0.002577 | 0.001802 |
| Methylobacterium | 0.000168 | 0.000911 | 0.000486 | 0.000412 | 0.000299 | 0.000479 | 0.000285 | 8.75E-05 | 0.000496 | 0.000866 | 0.002745 | 0.000767 |
| Micromonospora | 2.52E-05 | 8.76E-06 | 5.20E-05 | 5.26E-05 | 2.64E-05 | 0.000113 | 1.67E-05 | 4.37E-05 | 1.74E-05 | 7.72E-05 | 0.000602 | 7.14E-05 |
| Arthrobacter | 9.25E-05 | 7.89E-05 | 6.94E-05 | 9.64E-05 | 5.28E-05 | 8.71E-05 | 3.35E-05 | 8.75E-06 | 2.61E-05 | 8.58E-06 | 3.54E-05 | 0.000107 |
| Stenotrophomonas | 0 | 4.38E-05 | 2.60E-05 | 1.75E-05 | 3.52E-05 | 3.48E-05 | 8.37E-06 | 7.87E-05 | 4.35E-05 | 0.000163 | 0.000443 | 7.14E-05 |
| Paeniclostridium | 0 | 0 | 8.67E-06 | 0 | 0 | 0 | 0 | 1.75E-05 | 0 | 1.72E-05 | 0 | 0 |
| Rhodopseudomonas | 1.68E-05 | 3.50E-05 | 3.47E-05 | 2.63E-05 | 6.16E-05 | 8.71E-06 | 5.02E-05 | 2.62E-05 | 8.70E-06 | 0.000137 | 2.66E-05 | 8.92E-05 |
| Ensifer | 0 | 2.63E-05 | 8.67E-06 | 1.75E-05 | 0 | 1.74E-05 | 0 | 0 | 0 | 0 | 3.54E-05 | 8.92E-06 |
| Agromyces | 8.41E-06 | 0.000438 | 6.94E-05 | 2.63E-05 | 2.64E-05 | 0 | 0 | 0 | 5.22E-05 | 8.58E-06 | 1.77E-05 | 3.57E-05 |
| Acinetobacter | 0 | 4.38E-05 | 8.67E-06 | 8.77E-06 | 3.52E-05 | 0.000122 | 2.51E-05 | 8.75E-06 | 0 | 1.72E-05 | 1.77E-05 | 0 |
| Azorhizobium | 0 | 0 | 0 | 0 | 0 | 0 | 0 | 0 | 0 | 1.72E-05 | 0 | 0 |
| **Taxonomy** | **RRt1** | **RRt2** | **RRt3** | **RPRt1** | **RPRt2** | **RPRt3** | **RIRt1** | **RIRt2** | **RIRt3** | **RIPRt1** | **RIPRt2** | **RIPRt3** |
| Mycobacterium | 0.001539 | 0.003937 | 0.001156 | 0.000555 | 0.001014 | 0.000554 | 0.000847 | 0.000391 | 0.003785 | 0.002332 | 0.002391 | 0.001571 |
| Burkholderia-Caballeronia-Paraburkholderia | 0.018325 | 0.004122 | 0.003051 | 0.004103 | 0.021904 | 0.001239 | 0.002029 | 0.002267 | 0.007552 | 0.00293 | 0.003322 | 0.00319 |
| Devosia | 0.014991 | 0.008273 | 0.004188 | 0.009413 | 0.017058 | 0.013876 | 0.006029 | 0.013926 | 0.009848 | 0.014688 | 0.014836 | 0.01497 |
| Streptomyces | 0.028129 | 0.009404 | 0.0085 | 0.014511 | 0.010339 | 0.013231 | 0.024962 | 0.037321 | 0.028921 | 0.0142 | 0.016424 | 0.01283 |
| Mesorhizobium | 0.006657 | 0.003196 | 0.002274 | 0.005327 | 0.01088 | 0.001391 | 0.004728 | 0.003448 | 0.005119 | 0.004215 | 0.004204 | 0.004486 |
| Bradyrhizobium | 0.005 | 0.003732 | 0.005003 | 0.002747 | 0.010127 | 0.019771 | 0.003251 | 0.003201 | 0.006997 | 0.001724 | 0.002195 | 0.001914 |
| Bacillus | 9.80E-06 | 3.90E-05 | 5.69E-05 | 3.52E-05 | 3.86E-05 | 0 | 4.93E-05 | 0.000114 | 0.000175 | 9.96E-06 | 4.90E-05 | 2.94E-05 |
| Allorhizobium-Neorhizobium-Pararhizobium-Rhizobium | 0.130567 | 0.064286 | 0.048553 | 0.041394 | 0.086979 | 0.106748 | 0.083811 | 0.086388 | 0.051245 | 0.075144 | 0.071683 | 0.081596 |
| Pseudomonas | 0.002392 | 0.154307 | 0.044156 | 0.020261 | 0.003649 | 0.132394 | 0.02839 | 0.122823 | 0.006539 | 0.034249 | 0.034729 | 0.039993 |
| Methylobacterium | 0.025943 | 0.026632 | 0.032018 | 0.035556 | 0.028063 | 0.014753 | 0.012757 | 0.026586 | 0.026576 | 0.024762 | 0.024381 | 0.017582 |
| Micromonospora | 0.000176 | 2.92E-05 | 0 | 8.81E-06 | 0 | 6.05E-05 | 0.000177 | 0.000143 | 6.81E-05 | 0.000498 | 0.00049 | 0.000668 |
| Arthrobacter | 3.92E-05 | 1.95E-05 | 2.84E-05 | 1.76E-05 | 5.79E-05 | 0 | 0 | 0 | 3.89E-05 | 1.99E-05 | 8.82E-05 | 2.94E-05 |
| Stenotrophomonas | 0.000598 | 0.003313 | 0.000682 | 0.000255 | 0.000907 | 0.021837 | 0.000788 | 0.001572 | 0.000214 | 0.003537 | 0.002812 | 0.001669 |
| Paeniclostridium | 0 | 0 | 0 | 0 | 0 | 0 | 0 | 0 | 0 | 0 | 0 | 0 |
| Rhodopseudomonas | 0.001441 | 0.002719 | 0.001203 | 0.001321 | 0.003147 | 0.001401 | 0.000887 | 0.000524 | 0.000808 | 0.001923 | 0.00196 | 0.001472 |
| Ensifer | 0.000431 | 0.000175 | 6.63E-05 | 9.69E-05 | 0.000203 | 0.001058 | 0.000364 | 0.0002 | 0.000341 | 0.000548 | 0.000451 | 0.00055 |
| Agromyces | 3.92E-05 | 7.80E-05 | 7.58E-05 | 0.000229 | 5.79E-05 | 4.03E-05 | 1.97E-05 | 1.91E-05 | 0.000574 | 6.98E-05 | 8.82E-05 | 1.96E-05 |
| Acinetobacter | 0 | 0 | 0 | 8.81E-06 | 0 | 0 | 0 | 0 | 9.73E-06 | 0.000169 | 0.000255 | 0.000118 |
| Azorhizobium | 0.000137 | 7.80E-05 | 0.000133 | 8.81E-06 | 1.93E-05 | 1.01E-05 | 0.000187 | 0.000591 | 0.000117 | 0 | 1.96E-05 | 9.82E-06 |
| **Taxonomy** | **PRt1** | **PRt2** | **PRt3** | **PPRt1** | **PPRt2** | **PPRt3** | **PIRt1** | **PIRt2** | **PIRt3** | **PIPRt1** | **PIPRt2** | **PIPRt3** |
| Mycobacterium | 0.000736 | 0.000632 | 0.000442 | 0.000637 | 0.000688 | 0.00076 | 0.001092 | 0.00073 | 0.001023 | 0.000366 | 0.000458 | 0.000737 |
| Burkholderia-Caballeronia-Paraburkholderia | 0.014313 | 0.011924 | 0.001649 | 0.022185 | 0.021874 | 0.006253 | 0.016237 | 0.009144 | 0.006481 | 0.026948 | 0.003958 | 0.003673 |
| Devosia | 0.004842 | 0.010155 | 0.022944 | 0.010852 | 0.011291 | 0.010206 | 0.00788 | 0.008512 | 0.015729 | 0.013926 | 0.00503 | 0.01975 |
| Streptomyces | 0.010964 | 0.018385 | 0.032938 | 0.010216 | 0.009665 | 0.013808 | 0.204043 | 0.02283 | 0.032614 | 0.013595 | 0.007348 | 0.051854 |
| Mesorhizobium | 0.004378 | 0.00549 | 0.00918 | 0.007003 | 0.007323 | 0.007736 | 0.008824 | 0.009008 | 0.008509 | 0.003995 | 0.003482 | 0.003997 |
| Bradyrhizobium | 0.00354 | 0.005022 | 0.005439 | 0.007444 | 0.00761 | 0.010387 | 0.008674 | 0.00463 | 0.008689 | 0.002145 | 0.002923 | 0.005176 |
| Bacillus | 1.01E-05 | 3.67E-05 | 0 | 0 | 3.82E-05 | 6.65E-05 | 0.000448 | 5.84E-05 | 0 | 1.79E-05 | 9.16E-06 | 0.000108 |
| Allorhizobium-Neorhizobium-Pararhizobium-Rhizobium | 0.072764 | 0.073348 | 0.067013 | 0.139583 | 0.138509 | 0.130392 | 0.064202 | 0.168518 | 0.08488 | 0.158329 | 0.233439 | 0.095076 |
| Pseudomonas | 0.109308 | 0.043791 | 0.007491 | 0.025985 | 0.025105 | 0.039096 | 0.005275 | 0.006566 | 0.005117 | 0.002556 | 0.151454 | 0.05317 |
| Methylobacterium | 0.016935 | 0.029841 | 0.032446 | 0.032224 | 0.033939 | 0.017419 | 0.013679 | 0.025 | 0.017776 | 0.010207 | 0.005836 | 0.016391 |
| Micromonospora | 4.03E-05 | 1.83E-05 | 6.03E-05 | 0.000823 | 0.000889 | 0.000171 | 0.007684 | 0.000973 | 0.000426 | 3.58E-05 | 0.001576 | 0.003752 |
| Arthrobacter | 6.05E-05 | 6.42E-05 | 6.03E-05 | 2.94E-05 | 1.91E-05 | 1.90E-05 | 0.000168 | 5.84E-05 | 2.84E-05 | 8.94E-06 | 9.16E-06 | 4.91E-05 |
| Stenotrophomonas | 0.000363 | 0.000458 | 0.000473 | 0.00049 | 0.000325 | 0.000874 | 0.00014 | 2.92E-05 | 2.84E-05 | 0.000116 | 0.004783 | 0.000982 |
| Paeniclostridium | 0 | 0 | 0 | 0 | 0 | 0 | 0 | 0 | 0 | 0 | 0 | 0 |
| Rhodopseudomonas | 0.000171 | 0.000596 | 0.002544 | 0.001998 | 0.001836 | 0.001235 | 0.000336 | 0.001109 | 0.000445 | 0.001189 | 0.000376 | 0.000943 |
| Ensifer | 0.000373 | 0.000192 | 0.000483 | 0.000509 | 0.000822 | 0.000485 | 0.000112 | 0.000564 | 0.000417 | 0.008759 | 0.000907 | 0.000432 |
| Agromyces | 3.03E-05 | 4.58E-05 | 0.000131 | 0 | 0 | 0.000133 | 9.34E-06 | 6.81E-05 | 2.84E-05 | 4.47E-05 | 5.50E-05 | 3.93E-05 |
| Acinetobacter | 1.01E-05 | 0.00011 | 0.000161 | 0 | 0 | 0 | 0 | 1.95E-05 | 0 | 8.94E-06 | 0 | 0 |
| Azorhizobium | 2.02E-05 | 0 | 5.03E-05 | 9.79E-06 | 4.78E-05 | 0.000105 | 0 | 0 | 0 | 5.36E-05 | 3.66E-05 | 1.96E-05 |

Treatments’ details were as in Supplementary Table 3.
